# A novel dataset of North-Eastern Indian coins for machine learning-based classification

**DOI:** 10.1016/j.dib.2026.112813

**Published:** 2026-05-01

**Authors:** Ishtiak Al Mamoon, Saddat Kabir, Ariful Islam, Golam Moula, Wafial Hasnat Laisa, Abu Sufian, Md. Mahbub Hasan Akash, Sheekar Banerjee, Md. Samiul Alom, Md. Abdul Awal, A.K.M. Muzahidul Islam, Utpal Kanti Das

**Affiliations:** a Department of Computer Science and Engineering, International University of Business Agriculture and Technology, Dhaka, Bangladesh; b Department of Computer Science and Engineering, United International University, Dhaka, Bangladesh

**Keywords:** Numismatics, North eastern indian medieval coins, Dataset annotation, Ancient indian archaeology, Image processing

## Abstract

Old coins made by hand in ancient times and the Middle Ages are difficult to categorize because they are important for understanding culture and history, and they have complicated designs. The recent breakthroughs in machine learning have demonstrated strong potential for solving such classification tasks, but these approaches require well-structured datasets. This paper presents a new dataset of Indian coin images from the North-Eastern region, including Assam, Tripura, Koch Bihar, and Jaintiapur, which were historically part of the Bengali kingdoms. This dataset contains 51 classes and 2147 images. The images were collected from verified private collections and five well-established auction houses with permission. Each image showcases the ideal examples of these coins. They are usually difficult to recognize because they are very scarce and feature intricate designs and inscriptions. Each coin was carefully checked and confirmed using reliable numismatic sources, including the S. K. Bose numismatic series, verified auction house records, and authenticated private collections. The dataset includes images of extremely rare coins from the early 9th century AD to 1947, just before the partition of the Indian subcontinent. The historical, cultural, and antiquarian significance of these coins makes the dataset a valuable resource for study. It is anticipated that the methodologies will advance coin classification and make significant contributions to the archaeological research of ancient North-Eastern Indian history and culture.

Specifications Table

**Table:** 

Field	Description
Subject	Computer Science, archaeology, history.
Specific subject area	Numismatic data collection and classification using machine learning, focusing on North-Eastern Indian coins.
Types of data	Image; Annotation; Metadata
Data format	TIFF; XLSX
Data collection	The North-Eastern Indian coin images are collected from personal collections and five auction houses taking their permission. Images are in .tiff format with a size of 512 × 512 pixels. Collected data is cleaned and processed by the use of vector graphics processing software GNU, Inkscape, PIX. Data analysis is performed through a mix of visual analyses and experience with numismatic literature.
Data source location	Most images were obtained from private collections while additional coin images were collected from five auction houses that granted permission for their use. The data are validated by Ishtiak Al Mamoon (Life Member, Bangladesh Numismatic Collector’s Society BNCS-341)
Data accessibility	Repository name: North Eastern Indian Numismatics Data identification number: 10.17632/275grzntd3.3 Direct URL to data: https://data.mendeley.com/datasets/275grzntd3/3
Related research article	None

## 1 Value of the Data

• The collection consists of 2147 rare and precious coins across 51 classes of North-Eastern India, including both obverse and reverse sides that enable multi-class classification tasks in cultural heritage and numismatic research.
• The dataset can be reused by scholars in computer vision, machine learning, numismatics for tasks such as coin classification, fake coin detection, image retrieval, feature extraction, and domain adaptation across different regions and historical periods.
• This dataset supports practical applications including automated museum cataloging, auction house verification systems, and counterfeit detection workflows for North-Eastern Indian coinage.
• Detailed metadata annotation, such as king, dynasty, year, metal type, motif, and inscription, allows interdisciplinary research that combines archaeology, computer vision, and historical studies.
• The dataset provides a standardized benchmark for evaluating classification performance, cross-domain generalization, and transfer learning in historical image processing.

This dataset can be used for various machine learning tasks, including multi-class coin classification based on the North-Eastern Indian region, followed by dynasty or king-level categories. To preserve class distribution, researchers may use stratified sampling when splitting the dataset (e.g., 70 % training, 15 % validation, 15 % testing) following the standard supervised learning pipelines. For different classification tasks, convolutional neural network architectures, including ResNet or EfficientNet can be trained using the provided 512×512 images or resized inputs (e.g., 224 × 224). Moreover, the annotation files (CSV/XLSX) can be used to map image filenames to class labels and metadata that enable straightforward dataset loading and label encoding. During training, techniques such as class weighting, minority class oversampling, or data augmentation can be used to address class imbalance. For the fake coin detection task, this dataset can serve as a reference for authentic coin characteristics, enabling the learning of discriminating features such as inscriptions, motifs, and structural patterns. Additionally, a model trained on authentic samples can be used in anomaly detection settings, where deviations from learned feature distributions may indicate potential forgeries. From coin images, deep feature representations can be extracted for the image retrieval task and compared using similarity measures, such as cosine similarity or Euclidean distance, to retrieve visually similar coins. Furthermore, this dataset supports cross-region evaluation to assess generalization, where the model can be trained on one regional subset (e.g., Assam) and tested on other regions (e.g., Tripura or Koch Bihar). To facilitate dataset usage, initial exploration scripts are provided as supplementary materials. Additionally, for preparing inputs and extracting feature representations, our provided preprocessing and feature extraction scripts can serve as a baseline pipeline. It is important to clarify that the dataset is provided in its original, non-augmented form. Any augmentation, domain adaptation, or transfer learning strategies are meant for later use and are not part of the dataset preparation process.

## 2 Background

The creation of this dataset was motivated by two main objectives: preserving cultural heritage and advancing numismatic studies [1]. This work combines traditional numismatic knowledge with contemporary machine learning tools to develop a dataset focused on North-Eastern Indian coins [2,3]. Coins from different North-Eastern Indian regions, such as Assam, Tripura, Koch Bihar, and Jaintiapur, exhibit significant variations in design, motifs, inscriptions, and minting methods, which require systematic documentation for heritage and computational studies. The dataset can serve as an effective resource for machine learning-powered classification and analysis to gain a better understanding of North-Eastern Indian coinage in its cultural and historical context [[4], [5], [6], [7], [8]]. This study combines domain expertise with computational methodologies. As an independent data paper, this dataset offers a curated collection of coin images that can support further research in Indian history, archaeology, and artificial intelligence applications in numismatics [9,10]. Overall, this dataset contributes to cultural preservation and promotes data-driven research in heritage studies.

## 3 Data description

The dataset comprises four sections that represent different regions of the North-Eastern Indian area. The first section is devoted to Assam, which has two historical periods: The Pre-Ahom period and the Ahom period [11,12]. The Ahom period has 797 RGB images that represent eleven (11) different classes, whereas the pre-Ahom period has 74 such images that depict three (3) classes of Assamese coins. The second section covers Tripura, which contains 609 RGB images across eighteen (18) different classes of Tripura coins [13]. The third section is Koch Bihar, which comprises 421 RGB images representing fifteen (15) different classes of Koch Bihar coins [14]. The fourth section is centered on Jaintiapur, where 246 RGB images represent seven (7) classes of Jaintiapur coins [15]. The total number of coin images from four regions amounts to 2147 North-Eastern Indian coins across 51 classes that are stored in the TIFF format to ensure high visual quality. The complete dataset is publicly available in Mendeley Data [16].

Several recent datasets, including Roman imperial coin datasets, Ghana currency datasets, Indian coin image datasets, damaged Indian banknote currency, Bangladesh coin datasets, and Gupta Archer type coin dataset, have been introduced for numismatic and currency classification tasks [[4], [5], [6], [7], [8],^17^]. However, there is no publicly available dataset that majorly focuses on medieval and early modern North-Eastern Indian coinage at this scale and historical scope.

Each region also includes minor dynasty classes that group limited numbers of coin images under one or more dynasty classes, such as Assam (Minor Simha Dynasty), Tripura (Minor Manikya Dynasty I and II), Koch Bihar (Minor Narayana Dynasty I and II), and Jaintiapur (Minor Jaintiapur Dynasty). Furthermore, all images are provided as raw annotated data without any data augmentation.

The inclusion criteria of the images required that each coin should represent a particular dynasty or kingdom: Ahom Dynasty (Assam), Manikya Dynasty (Tripura), Narayana Dynasty (Koch Bihar), and Jaintia Kingdom (Jaintiapur). These classifications were confirmed by numismatic experts and validated using established numismatic references [1]. The sample is based on the coins of the four North-Eastern regions of India, where special motifs and other design elements make these coins distinct across different times and kingdoms. The major inclusion criterion was the presence of unique motifs and inscriptions that could support accurate classification by region, period, and dynasty. The coins contain inscriptions in Ahom, Bengali, Sanskrit, Arabic, and occasionally local variants, and they are made of copper, silver, and gold, providing a rich historical background for analysis and research. Photographs were excluded from the data set when they failed to identify the target coin types in a particular region, time, and dynasty. This ensures that only relevant coins from the region and kingdom are included in the dataset, thereby enabling reliable and accurate historical analysis. Table 1, Table 2, Table 3, Table 4, Table 5 provide a detailed overview of the North-Eastern Indian coin set, the names of each coin type, and the number of corresponding images. The images of the Pre-Ahom and Ahom-period, Tripura, Koch Bihar, and Jaintiapur are the obverse and reverse of a coin in the form of a square image.

**Table 1:** Description of Pre-Ahom coin dataset.

SN	Denomination Considered	No. of images	Acronyms
01	Harjaravarman	25	HV
02	Vanamalavarman	26	VV
03	Tyagasimha	23	TS
	**Total No of images**	**74**

**Table 2:** Description of Assam coin dataset.

SN	Denomination Considered	No. of images	Acronyms
01	Bharatha Simha	21	BRS
02	Brajanatha Simha	22	BJS
03	Chakradhvaja Simha	21	CS
04	Gadahara Simha	20	GDS
05	Gaurinatha Simha	278	GS
06	Lakshmi Simha	118	LS
07	Minor Simha Dynasty	22	MSD
08	Pramatta Simha	39	PS
09	Rajesvara Simha	97	RVS
10	Rudra Simha	40	RS
11	Siva Simha	119	SVS
	**Total No of images**	**797**

**Table 3:** Description of Tripura coin dataset.

SN	Denomination considered	No. of images	Acronyms
1	Amara Manikya	47	AM
2	Ananta Manikya	22	ATM
3	Deva Manikya	23	DeM
4	Dhanya Manikya	53	DM
5	Dharma Manikya	22	DhM
6	Govinda Manikya	21	GM
7	Jaya Manikya	22	JM
8	Kalyana Manikya	21	KM
9	Minor Manikya Dynasty I	21	MMD I
10	Minor Manikya Dynasty II	22	MMD II
11	Rajadhara Manikya	65	RajM
12	Rama Manikya	21	RMM
13	Ratna Manikya	35	RM
14	Udaya Manikya	20	UM
15	Vijaya Manikya	102	VM
16	Vira Chandra Manikya	21	VCM
17	Vira Vikrama Kishora Dev Barman	50	VVKDB
18	Yaso Manikya	21	YM
	**Total No of Images**	**609**

**Table 4:** Description of Koch Bihar coin dataset.

SN	Denomination considered	No. of images	Acronyms
1	Jagaddipendranarayana	23	JGN
2	Lakshminarayana	53	LN
3	Minor Narayana Dynasty I	23	MND I
4	Minor Narayana Dynasty II	20	MND II
5	Modanarayana	23	MN
6	Naranarayana	82	NN
7	Nirpendranarayana	21	NPN
8	Prananarayana	25	PN
9	Raghudevanarayana	22	RN
10	Rajendranarayana	23	RJN
11	Rajrajendranarayana	21	RRN
12	Rupanarayana	22	RPN
13	Shivendranarayana	22	SN
14	Upendranarayana	20	UN
15	Viranarayana	21	VN
	**Total No of Images**	**421**

**Table 5:** Description of Jaintiapur coin dataset.

SN	Denomination considered	No. of images	Acronyms
1	Bar Gosain II	98	BGII
2	Bijay Narayan	20	BN
3	Chattra Simha	20	CS
4	Minor Jaintiapur Dynasty	20	MJD
5	Jatra Narayan	20	JN
6	Jay Narayan	20	JYN
7	Ram Simha II	48	RSII
	**Total No of Images**	**246**	

Table 1 summarizes the total number of images for each of the denominations of the Assam Pre-Ahom period along with the acronyms and names assigned for each of the denominations.

**Fig. 1:**
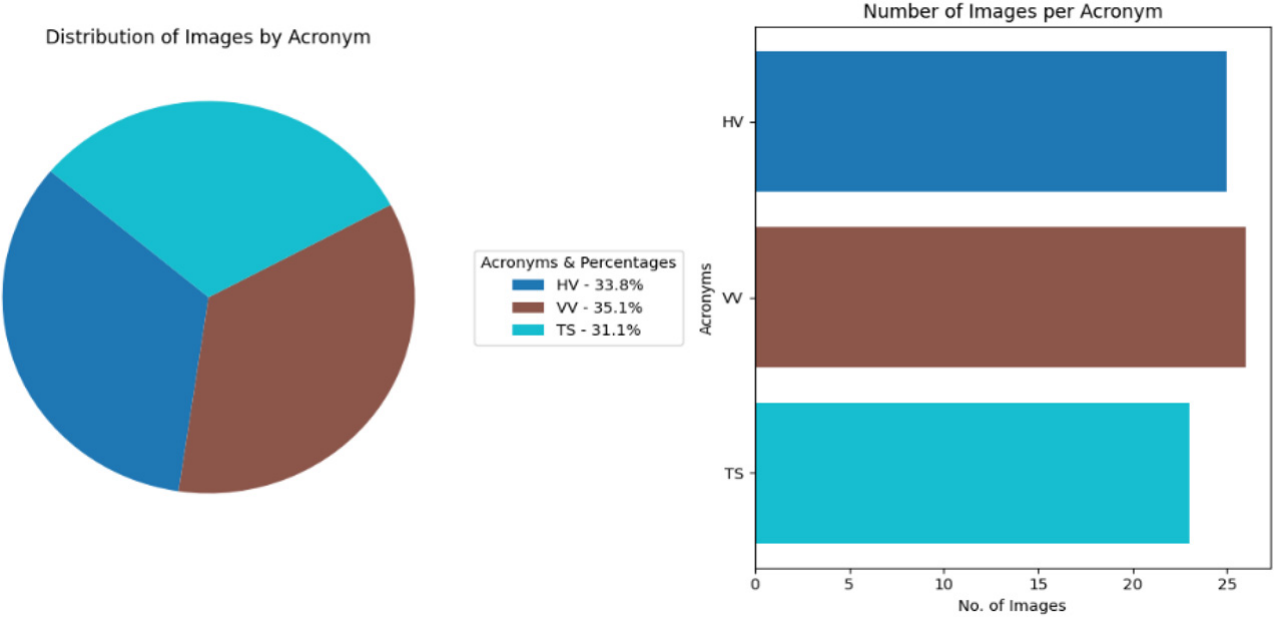
Distribution of images per class and No of image in each denomination in the Pre-Ahom type coin dataset.

**Fig. 2:**
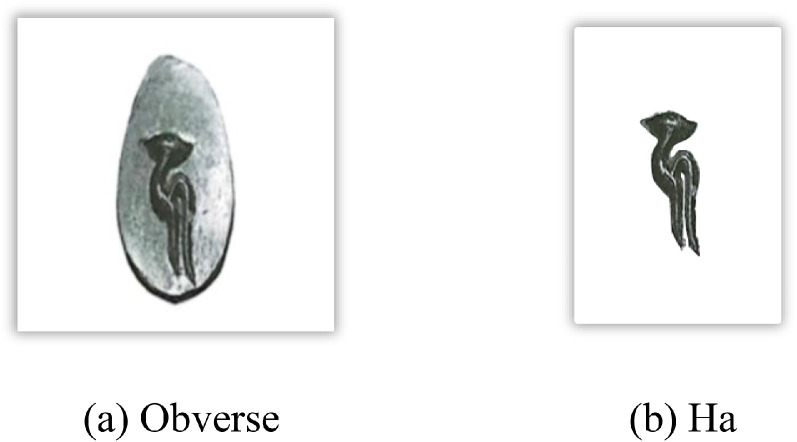
Important features of the Assam’s Pre-Ahom coin.

Fig. 1 visually represents the distribution of Assam’s Pre-Ahom period coins, each denomination, and number of images per class in the dataset, and Fig. 2 shows the important features of the obverse a sample Pre-Ahom period coin image, specifically, from king Harjaravarman. In the North-Eastern Indian’s Pre-Ahom period coins, on the obverse surface of the coin, there is only one symbol (such as Ha, VA, and TA).

Table 2 summarizes the total number of images for each of the denominations of the Assam period, along with the acronyms and names assigned for each of the denominations.

**Fig. 3:**
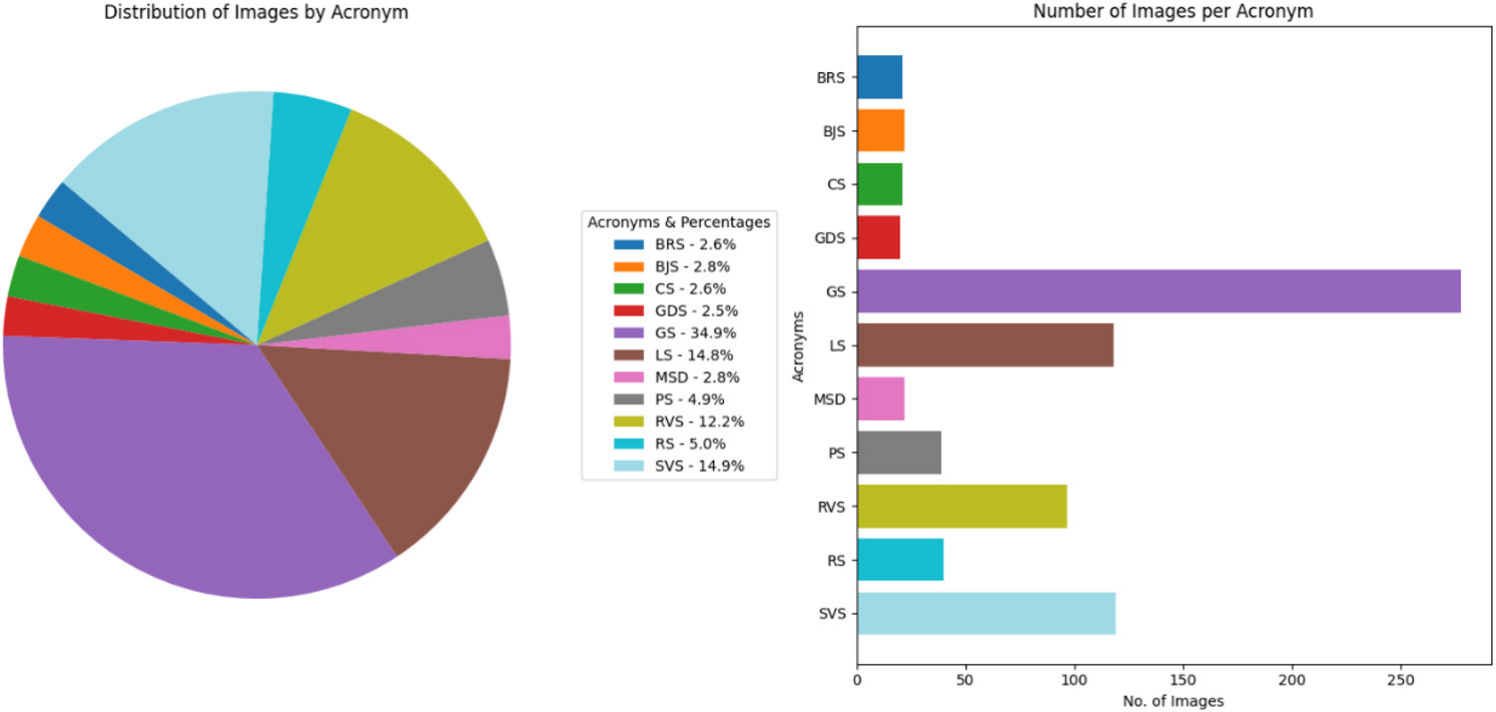
Distribution of images per class and No of images each denomination in the Assam coin dataset.

**Fig. 4:**
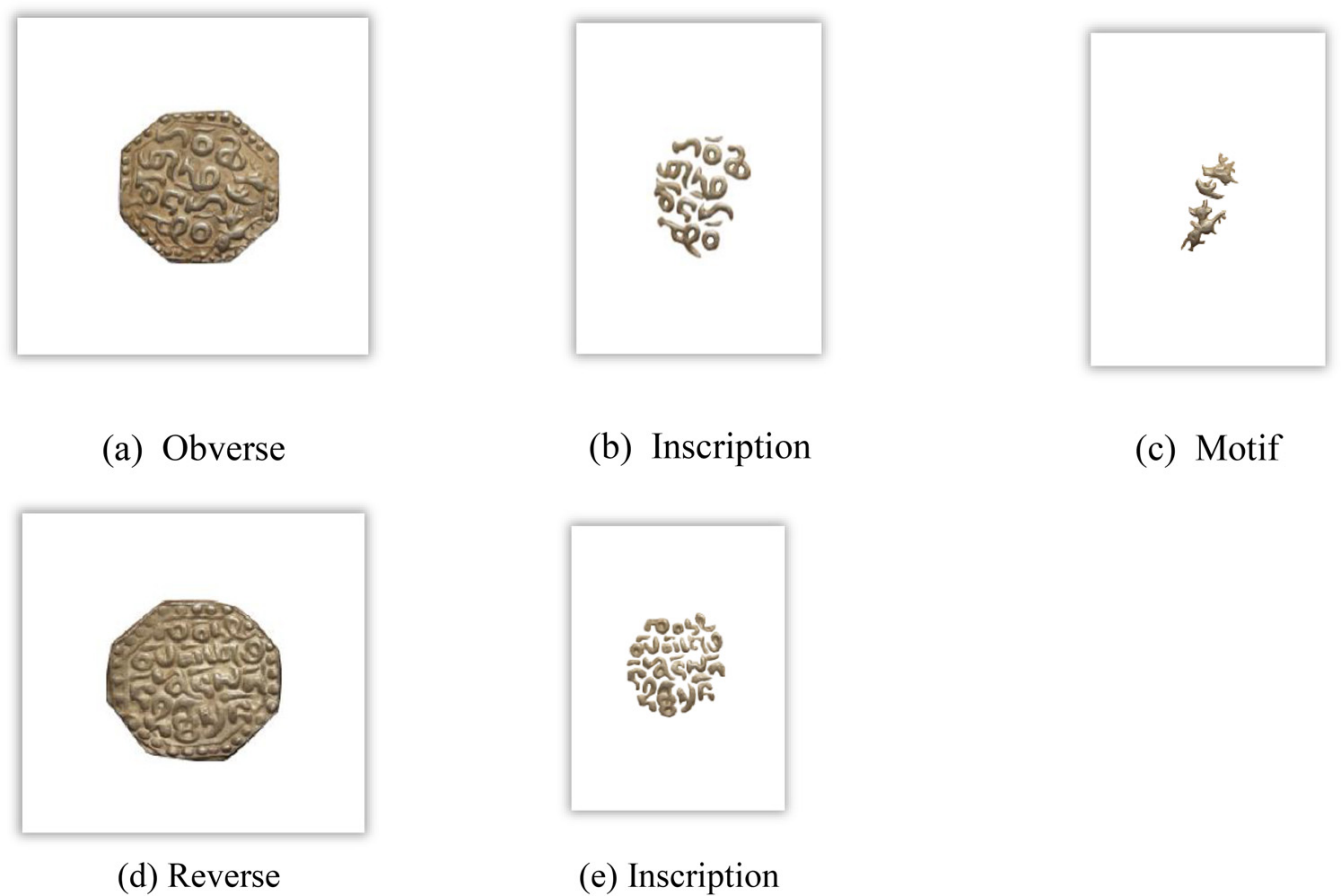
Important Feature of Brahmi Assam coin.

**Fig. 5:**
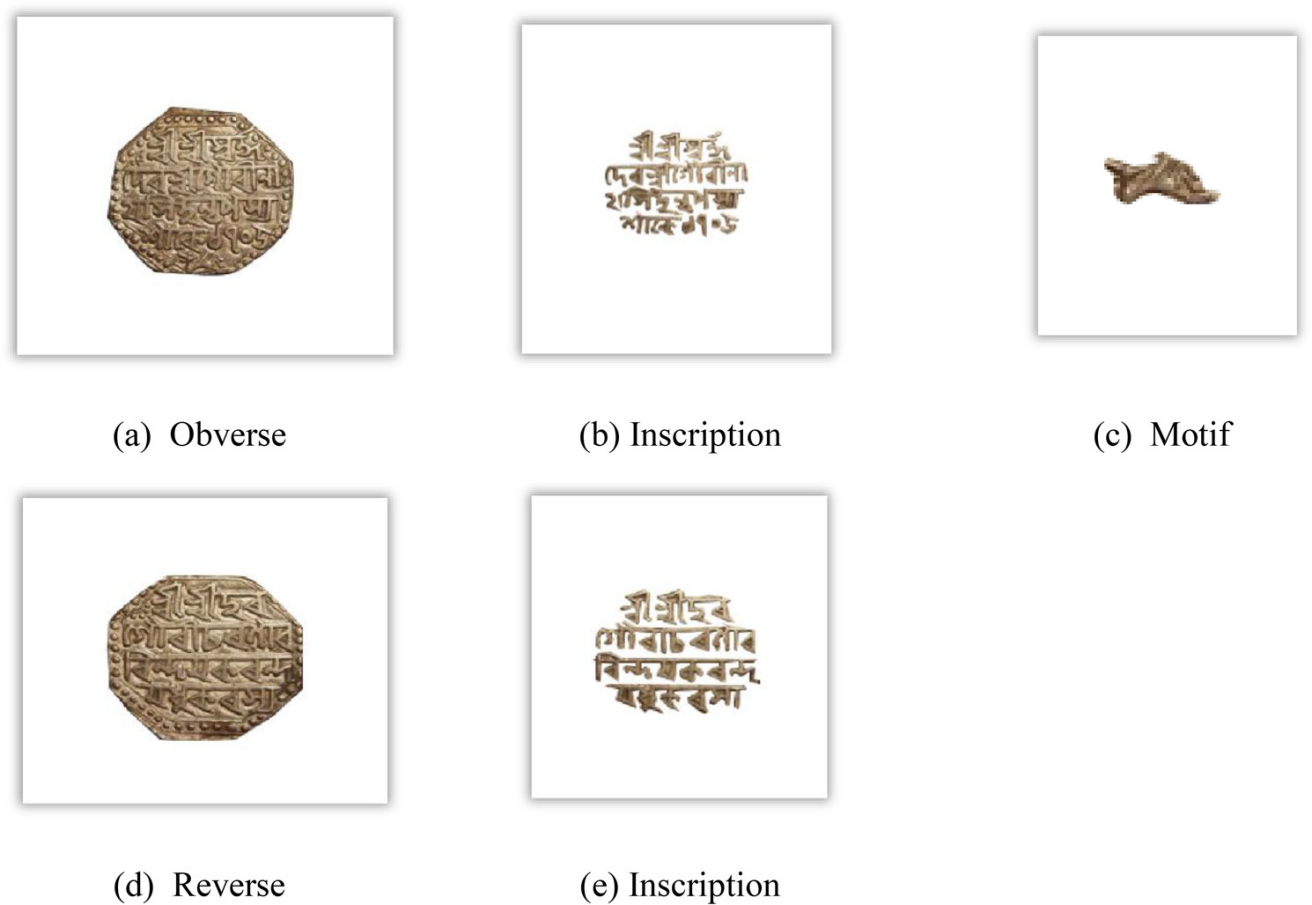
Important features of the Assamese Assam coin.

**Fig. 6:**
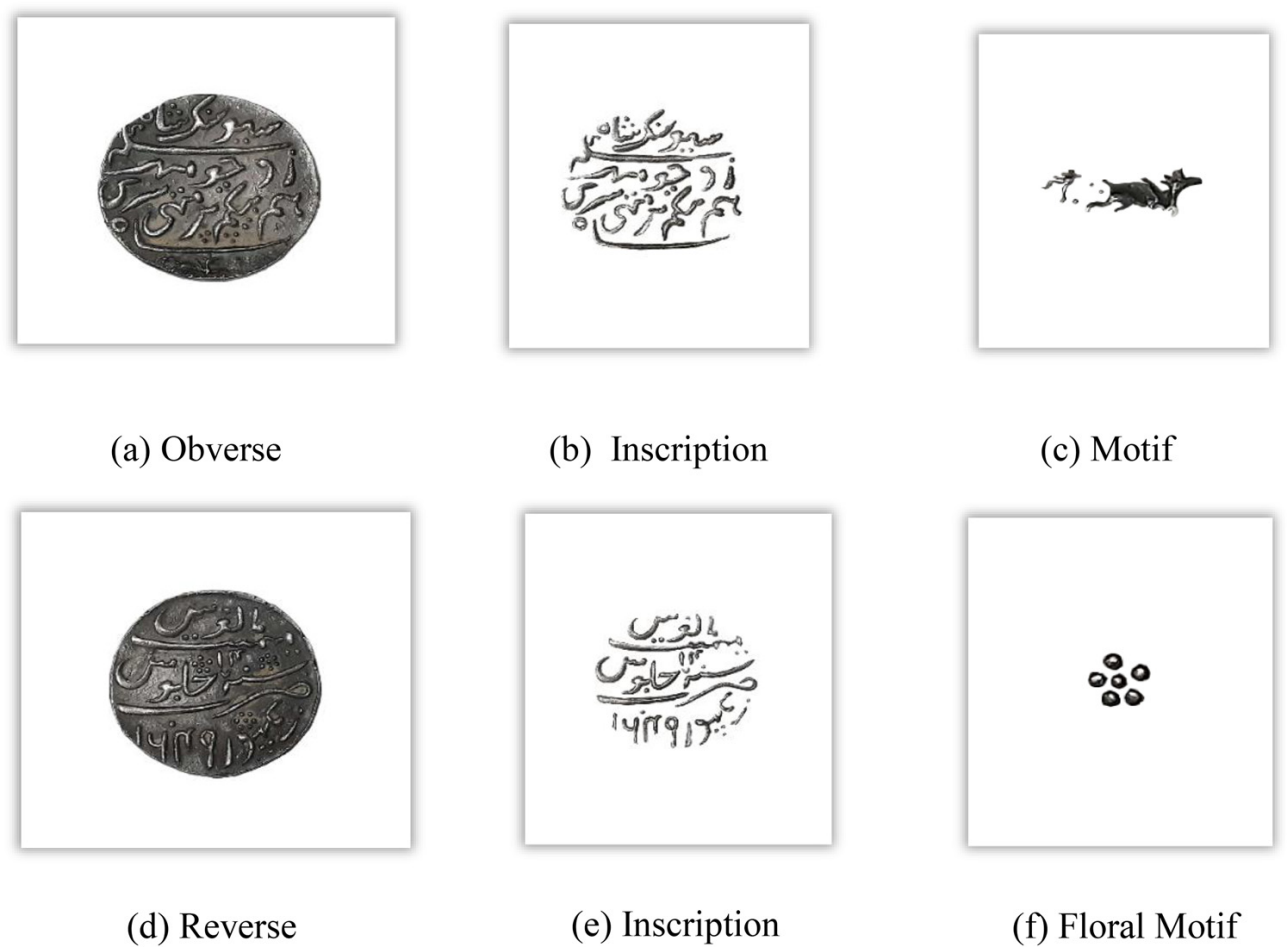
Important features of the Arabic Assam coin.

Fig. 3 visually represents the distribution of Assam coin classes, denomination, and number of images per class in the dataset. Figs. 4, 5, and 6 reveal the main coins of the three languages on the obverse and reverse, while the segmented parts, including inscription and motif, are displayed in other subpoints. These examples align with the sample images of Assam coins and include the Brahmi script of King Gadahara Simha, King Gaurinatha simha (Assamese), and king Siva simha (Arabic). Inscriptions on the obverse side of the Ahom coins include the king's name and symbols of birds and lions, whereas on the reverse side, inscriptions include symbols of the crescent moon, lion figures, and dot-patterned floral designs.

Table 3 summarizes the total number of images for each of the denominations of the Tripura coin dataset, along with the acronyms and names assigned for each of the denominations.

**Fig. 7:**
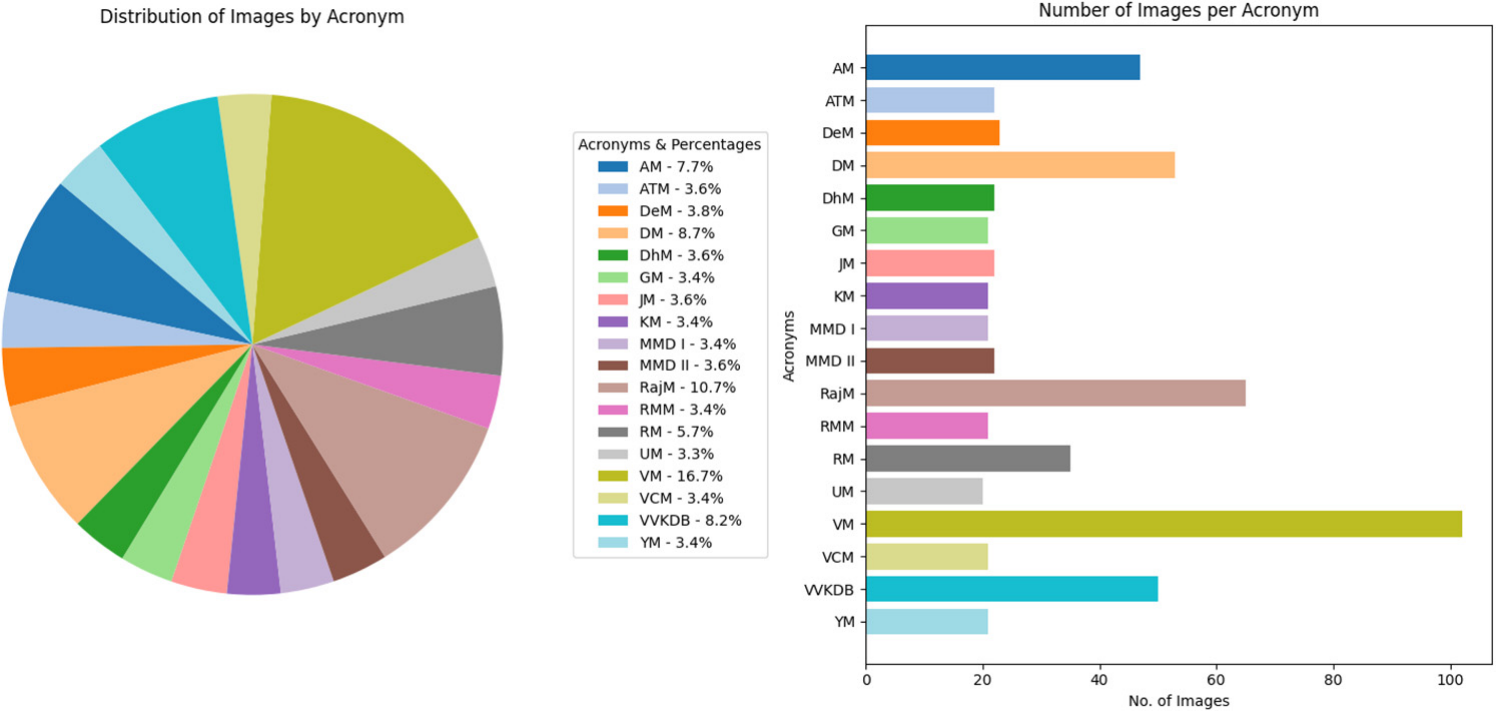
Distribution of each coin denomination and number of images per class in the Tripura coin dataset.

**Fig. 8:**
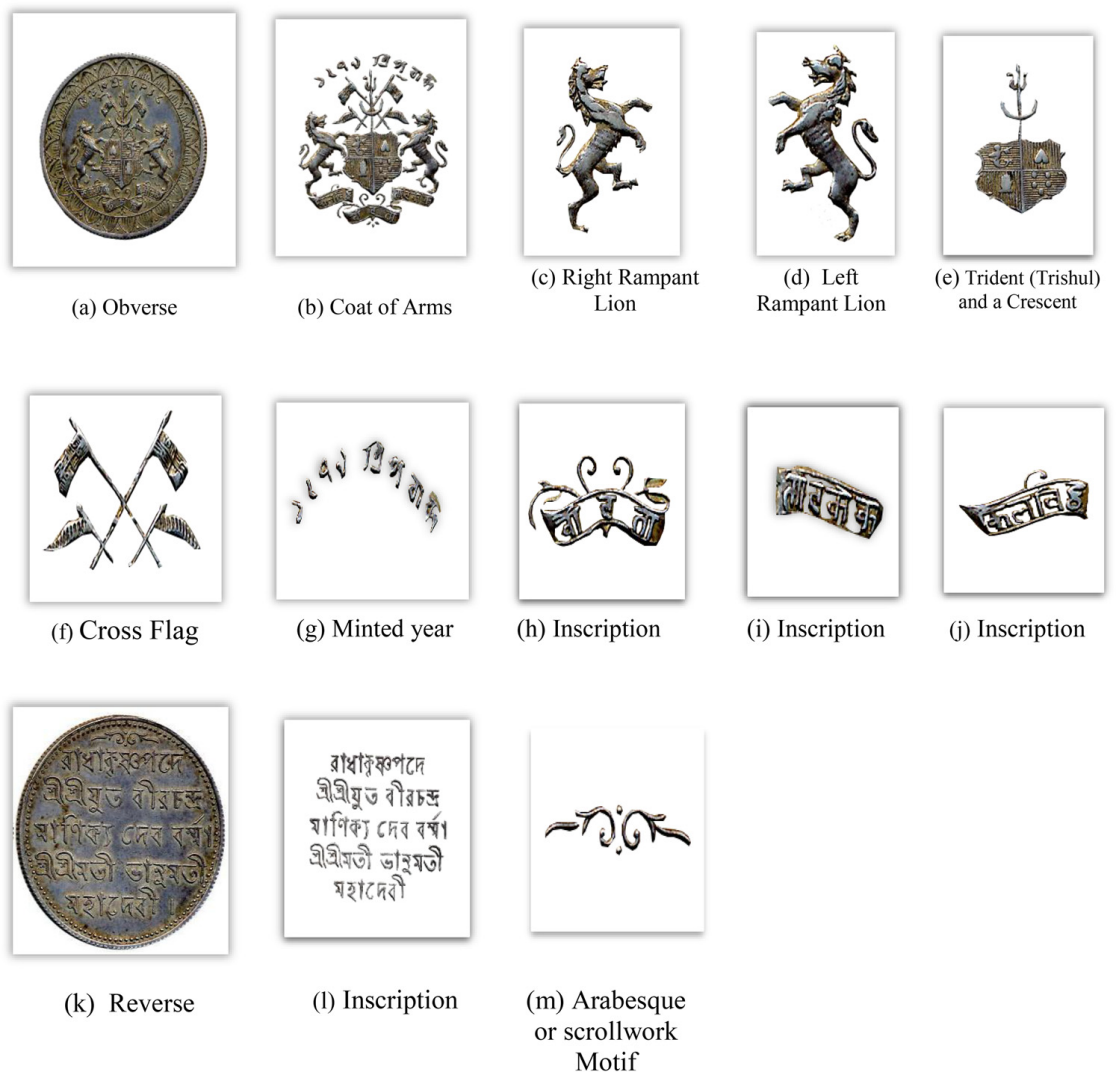
Important features of a Tripura coin.

Fig. 7 is a visual representation of the distribution of the Tripura coins in each denomination and number of images per class, and Fig. 8 displays the important features on the obverse and reverse sides of a sample Tripura coin image, i.e., a coin of Vira Chandra Manikya. The inscriptions on the faces of Tripura coins feature motifs of animals and floral motifs, and sometimes the date of issue is also indicated. The back features inscriptions of the king, arabesque patterns, and more.

Table 4 summarizes the total number of images for each of the denominations of the Koch Bihar coin dataset along with the acronyms’ names assigned for each of the denominations.

**Fig. 9:**
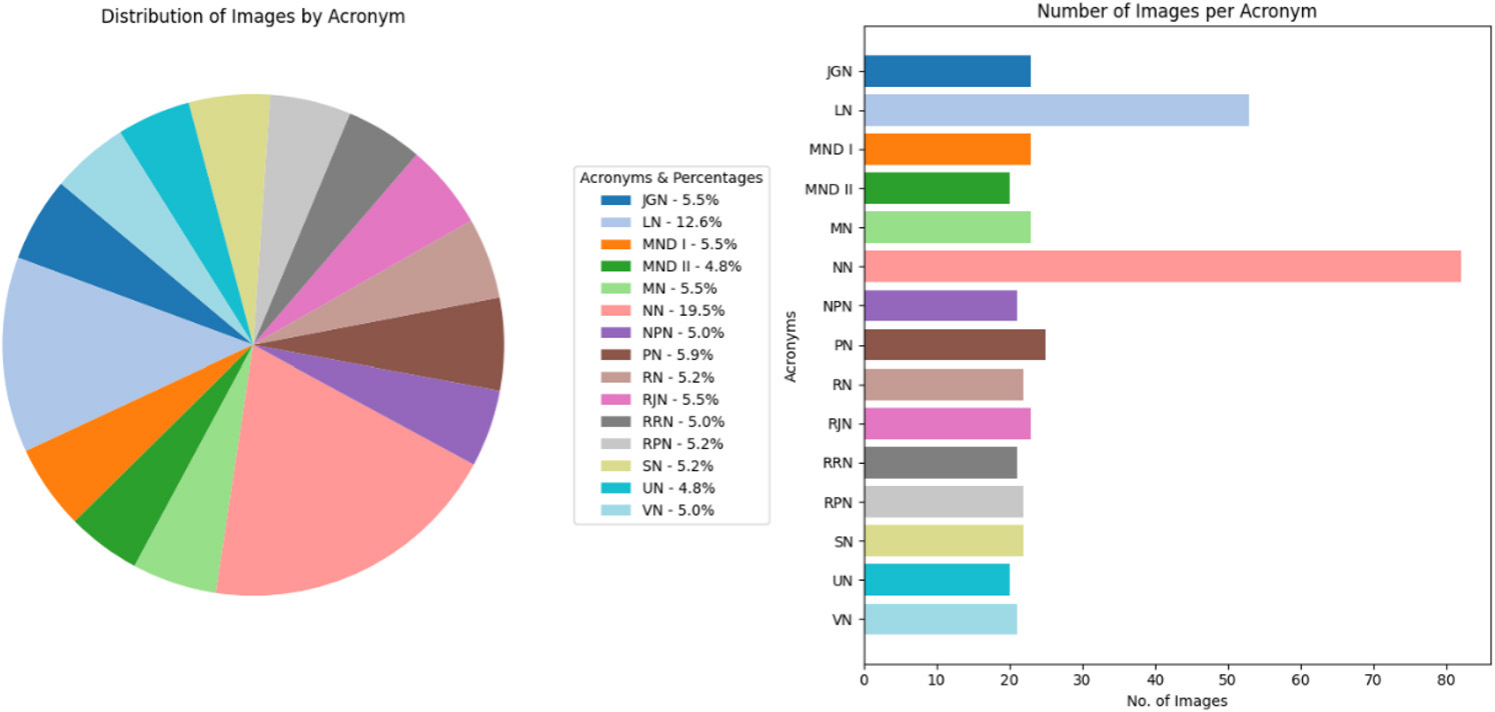
Distribution of each coin denomination and number of images per class in the Koch Bihar coin dataset.

**Fig. 10:**
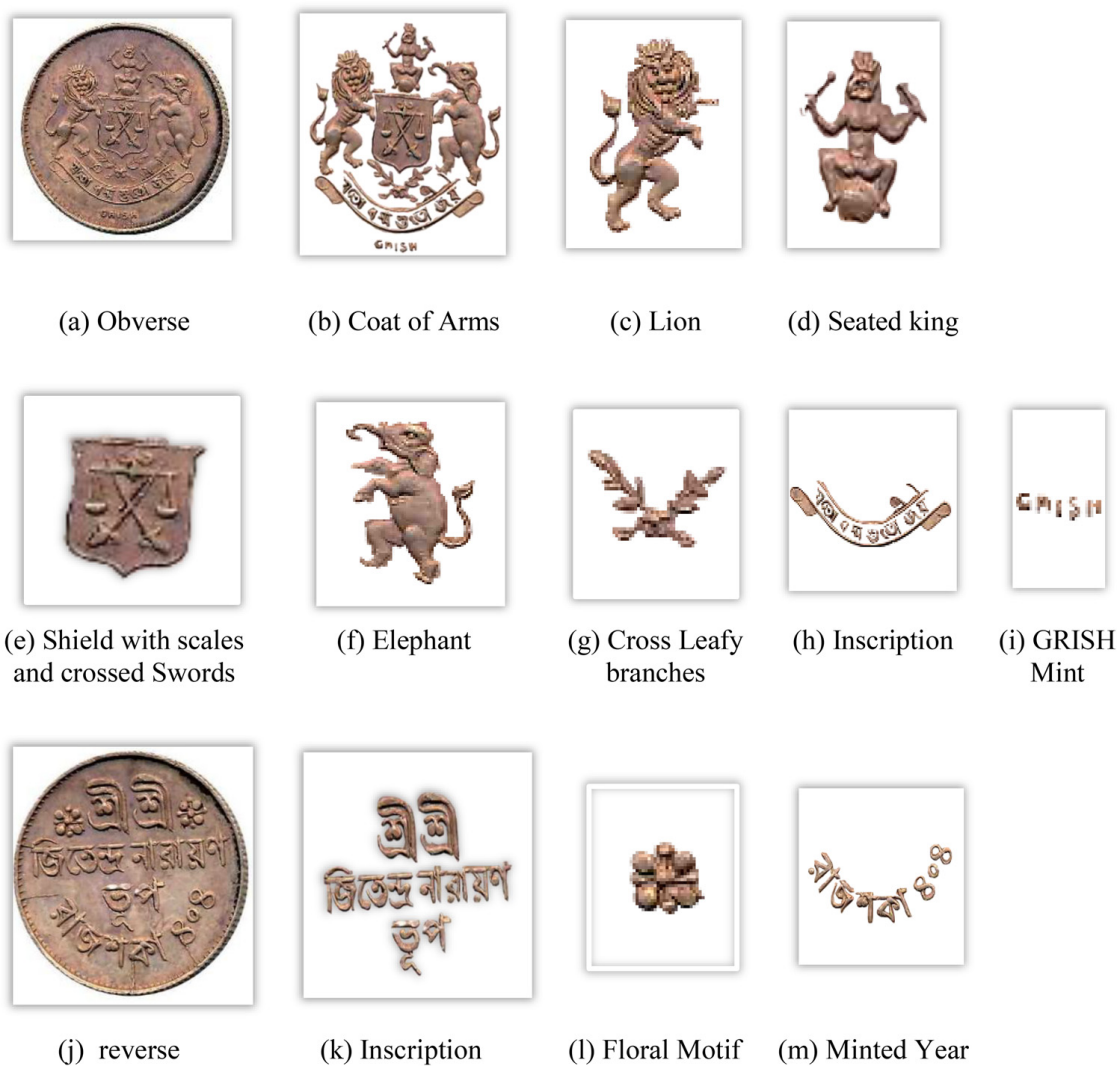
Important features of a Koch Bihar coin.

Fig. 9 visually displays the distribution of Koch Bihar coins by denomination and the number of images per classification in the dataset, and Fig. 10 displays the key features of a sample Koch Bihar coin image, namely, King Jitendra Narayan. The obverse side of Koch Bihar coins is usually decorated with the state's coat of arms, supported by a lion and an elephant; some coins bear inscriptions with different motifs. Although the reverse usually bears the king's inscriptions with a floral pattern, in certain instances, it also bears the year of issue.

Table 5 summarizes the total number of images for each of the denominations of the Jaintiapur coin dataset along with the acronyms' names assigned for each of the denominations.

**Fig. 11:**
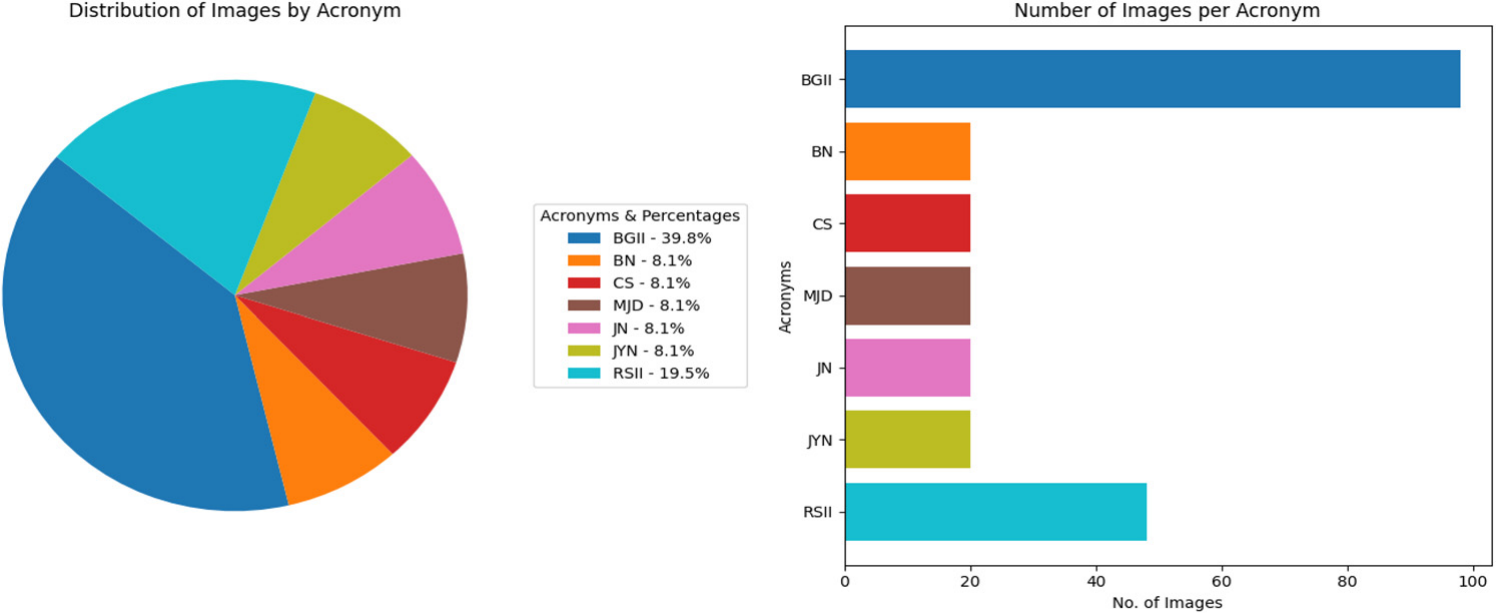
Distribution of each coin denomination and number of images per class in the Jaintiapur coin dataset**.**

**Fig. 12:**
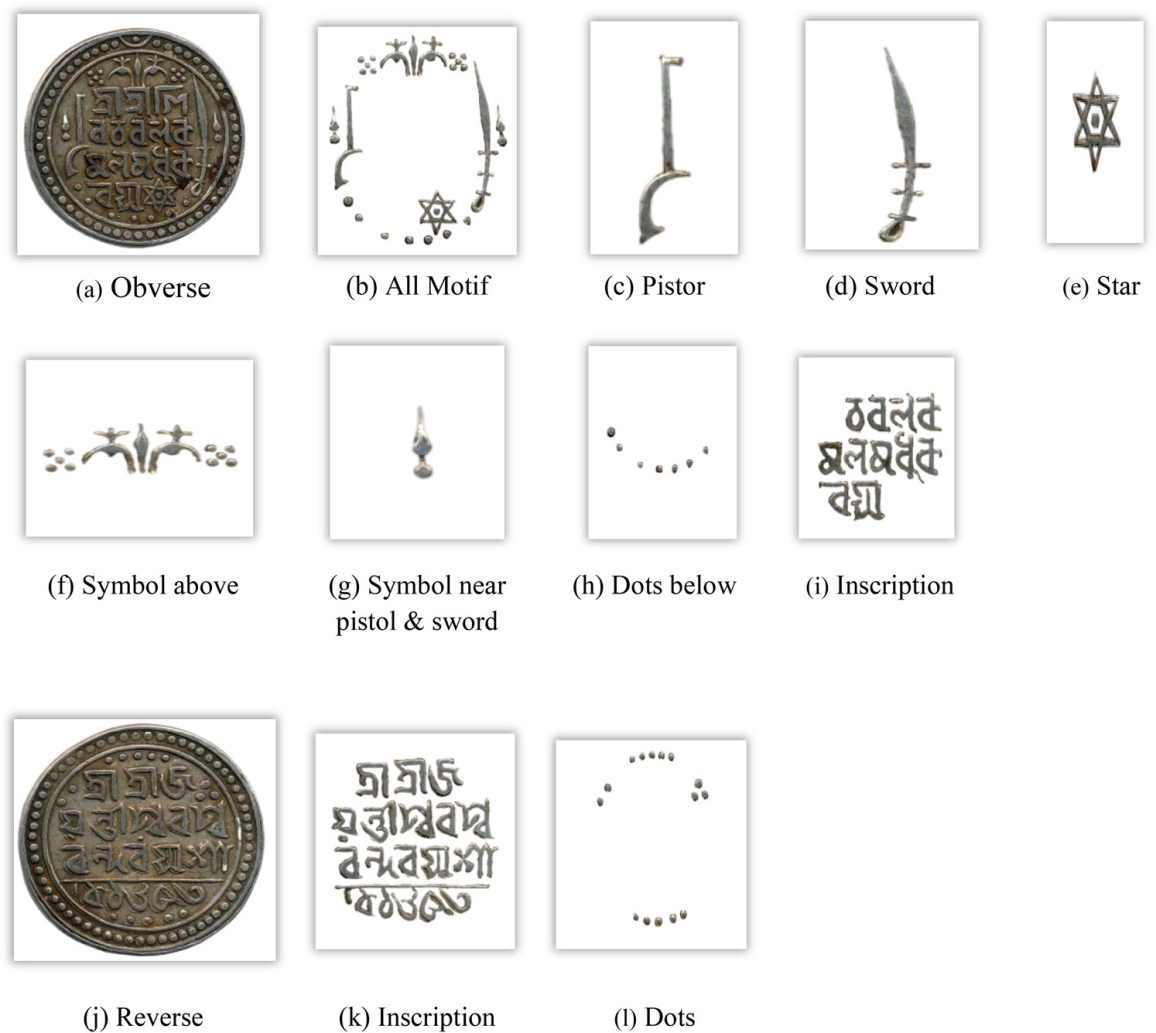
Important features of a Jaintiapur coin.

Fig. 11 visually displays the distribution of the Jaintiapur coins in each denomination and the number of images in each class within the data, and Fig. 12 indicates the significant features on both sides of a representative image of a Jaintiapur coin using a sample coin of King Bar Gosain II. Jaintiapur coins bear the king's inscription on their obverse, along with symbols such as the Star of David, a pistol, a sword, and others, which appear on some of them. The back has inscriptions and either floral dots, dots in an arc, or the date of issue. Table 6 summarizes all 51 classes in the North-East Indian coin dataset, including their region, corresponding period or dynasty, acronym, and total number of images.

**Table 6:** Comprehensive summary of all 51 coin classes across four regions.

Region	Period/Dynasty	Class Name	Acronym	No. of Images
Assam	Pre-Ahom	Harjaravarman	HV	25
Assam	Pre-Ahom	Vanamalavarman	VV	26
Assam	Pre-Ahom	Tyagasimha	TS	23
Assam	Ahom	Bharatha Simha	BRS	21
Assam	Ahom	Brajanatha Simha	BJS	22
Assam	Ahom	Chakradhvaja Simha	CS	21
Assam	Ahom	Gadahara Simha	GDS	20
Assam	Ahom	Gaurinatha Simha	GS	278
Assam	Ahom	Lakshmi Simha	LS	118
Assam	Ahom	Minor Simha Dynasty	MSD	22
Assam	Ahom	Pramatta Simha	PS	39
Assam	Ahom	Rajesvara Simha	RVS	97
Assam	Ahom	Rudra Simha	RS	40
Assam	Ahom	Siva Simha	SVS	119
Tripura	Manikya	Amara Manikya	AM	47
Tripura	Manikya	Ananta Manikya	ATM	22
Tripura	Manikya	Deva Manikya	DeM	23
Tripura	Manikya	Dhanya Manikya	DM	53
Tripura	Manikya	Dharma Manikya	DhM	22
Tripura	Manikya	Govinda Manikya	GM	21
Tripura	Manikya	Jaya Manikya	JM	22
Tripura	Manikya	Kalyana Manikya	KM	21
Tripura	Manikya	Minor Manikya Dynasty I	MMD I	21
Tripura	Manikya	Minor Manikya Dynasty II	MMD II	22
Tripura	Manikya	Rajadhara Manikya	RajM	65
Tripura	Manikya	Rama Manikya	RMM	21
Tripura	Manikya	Ratna Manikya	RM	35
Tripura	Manikya	Udaya Manikya	UM	20
Tripura	Manikya	Vijaya Manikya	VM	102
Tripura	Manikya	Vira Chandra Manikya	VCM	21
Tripura	Manikya	Vira Vikrama Kishora Dev Barman	VVKDB	50
Tripura	Manikya	Yaso Manikya	YM	21
Koch Bihar	Narayana	Jagaddipendranarayana	JGN	23
Koch Bihar	Narayana	Lakshminarayana	LN	53
Koch Bihar	Narayana	Minor Narayana Dynasty I	MND I	23
Koch Bihar	Narayana	Minor Narayana Dynasty II	MND II	20
Koch Bihar	Narayana	Modanarayana	MN	23
Koch Bihar	Narayana	Naranarayana	NN	82
Koch Bihar	Narayana	Nirpendranarayana	NPN	21
Koch Bihar	Narayana	Prananarayana	PN	25
Koch Bihar	Narayana	Raghudevanarayana	RN	22
Koch Bihar	Narayana	Rajendranarayana	RJN	23
Koch Bihar	Narayana	Rajrajendranarayana	RRN	21
Koch Bihar	Narayana	Rupanarayana	RPN	22
Koch Bihar	Narayana	Shivendranarayana	SN	22
Koch Bihar	Narayana	Upendranarayana	UN	20
Koch Bihar	Narayana	Viranarayana	VN	21
Jaintiapur	Jaintia Kingdom	Bar Gosain II	BGII	98
Jaintiapur	Jaintia Kingdom	Bijay Narayan	BN	20
Jaintiapur	Jaintia Kingdom	Chattra Simha	CS	20
Jaintiapur	Jaintia Kingdom	Minor Jaintiapur Dynasty	MJD	20
Jaintiapur	Jaintia Kingdom	Jatra Narayan	JN	20
Jaintiapur	Jaintia Kingdom	Jay Narayan	JYN	20
Jaintiapur	Jaintia Kingdom	Ram Simha II	RSII	48
		**Total No of Images**		**2147**

The class distribution of this dataset is presented separately by region in Table 1, Table 2, Table 3, Table 4, Table 5, along with corresponding pie and bar charts in Figs. 1, 3, 7, and 9, which visualize the coin distribution and the number of images per class. Additionally, Table 6 presents a combined summary of all 51 classes from four regions. The dataset exhibits a non-uniform distribution, where certain classes contain relatively more coin images. On the other hand, some classes have fewer images, leading to a moderately imbalanced dataset. Some dominant classes include >100 images, and several minor classes have around 20 images.

This imbalance mainly occurs due to historical minting patterns and ruling durations of different kings. Kings who ruled for longer periods minted more coins, so their coins appear more frequently in the dataset. In contrast, kings with short ruling periods minted fewer coins, and their coins are less common in the dataset. For example, Gaurinatha Simha has a ruling period of 16 years (1780 – 1796 CE), so his coins are more frequent (278 images) in the dataset, while other kings with short ruling periods, such as Brajanatha Simha (1818 – 1819 CE), are presented by fewer coins (21 images). Therefore, this imbalance reflects the historical distribution of coins rather than how data was collected. Furthermore, during dataset construction, classes with very limited samples were grouped into minor dynasty categories to reduce extreme class imbalance. However, this dataset still maintains a moderate class imbalance, which should be considered during the model development phase.

To address class imbalance, appropriate strategies such as class weighting, oversampling of minority classes, and data augmentation can be applied during model training to improve generalization. Moreover, stratified train-test splitting is recommended during evaluation to preserve the class distribution. When applying classification and recognition models to the dataset, these approaches can help ensure robust, unbiased performance.

## 4 Experimental Design, Materials and Methods

### 4.1 Experimental Design

Out of the 2147 images of the North-Eastern Indian country 1220 images were collected from our own museum collection. Five of the popular auction houses, i.e., Marudhar Arts, Oswal Auction, Classical Numismatic Gallery, David Feldman, and JAIN AUCTION HOUSE, have allowed the usage of 215, 207, 195, 115, and 195 images, respectively. In line with their requests, we have appended acronyms of the images obtained in Marudhar Arts, Oswal Auction House, Classical Numismatic Gallery, David Feldman, and JAIN Auction House as per their direction to the copyright name of the corresponding images.

Table 7 provides an overview of the distribution of coins collected from different sources, showing coin numbers for each auction house and private source. The total number of Assam coin images (871) represents the combined count of the Pre-Ahom (74 images) and Ahom (797 images) periods. All images sourced from private collections were captured with an iPhone 15 Pro Max, and the camera specifications are listed in Table 8. The images obtained are separated into classes and subclasses. Annotation of the coins was carried out on a Google spreadsheet. The final annotation files are provided in Excel and CSV format and stored in the Mendeley root directory [16].

**Table 7:** Coin distribution according to sources.

Source	Marudhar Arts	Oswal Auction House	Classical Numismatic Gallery	Jain Auction House	David Feldman	Private	Total
Assam (Combined: Pre-Ahom + Ahom)	72	83	62	75	10	569	871
Tripura	67	66	71	50	73	282	609
Koch Bihar	45	36	36	47	20	237	421
Jaintiapur	31	22	26	23	12	132	246
**Total**	215	207	195	195	115	1220	**2147**

**Table 8:** Camera Specification.

Device	Description
Camera	iPhone 15 Pro Max
Type	Smartphone
Megapixel	48 MP

Table 8 summarizes the camera specifications used for image acquisition, along with the device name and the camera sensor. The coin collection process started in June 2025 and continued through January 2026. The process to make the annotation commenced in April 2025, and the whole process was completed in January 2026. Table 9 gives a detailed description of data set collection (tasks and timeline).

**Table 9:** Data collection: Tasks and timeline.

SN	Task	Duration
01	Capture and collection of images	June 2025- January 2026
02	Image cleaning and preprocessing	August 2025- January 2026
03	Removal of background and image resize	September 2025- January 2026
04	Image annotation	June 2025– January 2026

## 5 Materials and Methods

Original photographs have been taken with different resolutions of 505×277, 655×350, 600×450, 483×278, 777×450, and 800×550 on different backgrounds as well.

### 5.1 Data Cleaning and Preprocessing

A Python-based pipeline with OpenCV, NumPy, and PIL libraries was used to process all the collected raw images. Firstly, for reducing image noise, a bilateral filtering technique was applied with parameters (*d* = 9, sigmaColor = 75, sigmaSpace = 75). This method preserves important edge details such as inscription, motif, and coin boundaries and smooths minor noise. Secondly, background normalization was performed where the background color was estimated through sampling small patches (20×20 pixels) from all four corners of images. The average pixel value of these corner regions was used as the background reference color, and the Euclidean distance between each pixel value and the estimated background color was calculated for every pixel in the image. Pixels with a difference value lower than 40 were considered background and replaced with pure white RGB values (255, 255, 255). Thirdly, the LANCZOS resampling method was applied to resize all the images to 512×512 pixels. This method preserves fine visual details during down sampling and also maintains the sharpness of engraved features. Finally, processed images were saved in TIFF format to maintain higher visual quality and avoid compression artifacts. Furthermore, no data augmentation, artificial enhancement techniques, color correction, or lighting normalization were applied throughout the whole process. The goal was to preserve the authenticity of the original coins to ensure consistency across the dataset. However, users may use such techniques during downstream machine learning tasks, depending on the specific application requirements. All preprocessing operations were implemented using Python 3.10 with OpenCV 4.8, NumPy 1.26, and Pillow (PIL) 10.0 libraries.

To make the dataset visually consistent while preserving the original details of the coin, selected preprocessing steps were applied. We used bilateral filtering to reduce minor image noise without blurring important features such as the inscription and motif. Different photography conditions produce variations; background normalization was applied to minimize those that could prevent the background pattern from influencing machine learning models. All images were resized to 512×512 pixels, ensuring a uniform square image size across the entire dataset. For deep learning-based models, this resolution preserves fine visual details while keeping computational costs manageable. This fixed square format ensures compatibility with convolutional neural network architectures and supports consistent benchmarking. Overall, these steps preserve the authenticity of the coin images while improving their suitability for machine learning applications.

### 5.2 Feature Extraction and Segmentation Procedure

A structured feature extraction procedure was implemented using OpenCV with Python and PyTorch libraries to visually illustrate important coin features such as inscriptions and motifs (Figs. 2, 4, 5, 6, 8, 10, and 12). Firstly, circular detection was used to separate coin regions from the background. The grayscale coin image was smoothed using median filtering (kernel size = 5), and the coin boundary was detected using the Hough circle transform with parameters (dp = 1.2, minDist = 100, param1 = 50, param2 = 30, minRadius = 50). After that, a binary mask was generated to separate the coin region from the background. In case circular detection failed, an alternative contour-based thresholding approach was used as a fallback to detect the largest connected component of the coin. This feature extraction procedure was applied to a limited number of images (Figs. 2, Fig. 4, Fig. 5, Fig. 6, Fig. 7, Fig. 8, 10, and 12) for methodological demonstration, rather than the entire dataset. In these demonstrated cases, the Hough Circle Transform failed to reliably detect the coin boundary for two representative samples: one non-circular elongated coin (Fig. 2) and one worn coin with weak boundary contrast (Fig. 6), where contour-based thresholding was required as a fallback approach. This ensured robust segmentation across different coin shapes and image conditions. After background removal, internal visual components were extracted, and edge detection (Canny operator with threshold values of 50 and 150) was applied within the masked coin region to highlight engraved inscriptions and motifs. Additionally, small noisy contours were filtered using an area threshold of 200 pixels. To isolate significant sub-regions corresponding to inscription and decorative elements, bounding rectangles were applied.

Finally, for deep feature representation, a pre-trained ResNet-50 convolutional neural network (Feature_Extraction part 3) was used to process segmented coin images. The final classification layer was removed, and the output of the last pooling layer was extracted as a feature vector that generates a fixed-length numerical representation of the coin image. Before passing to the neural network, all the input images were resized to 224×224 pixels. All the related Python scripts (Feature_Extraction part 1, Feature_Extraction part 2, and Feature_Extraction part 3) used for the feature extraction process are provided as supplementary materials. Moreover, the feature extraction process was implemented using Python 3.10 with OpenCV 4.8 and PyTorch 2.0 libraries.

### 5.3 Annotation structure and file organization

**Fig. 13:**
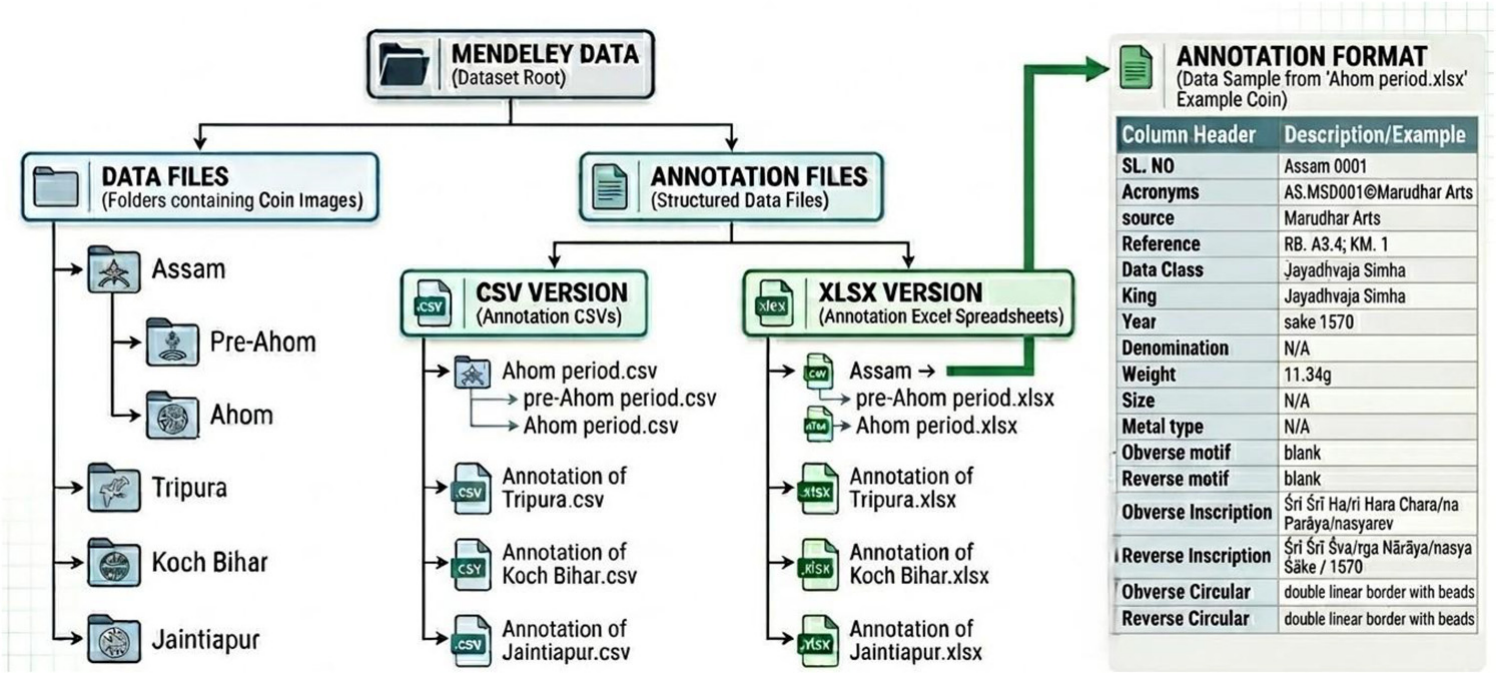
Overall dataset structure and annotation file organization.

**Fig. 14:**
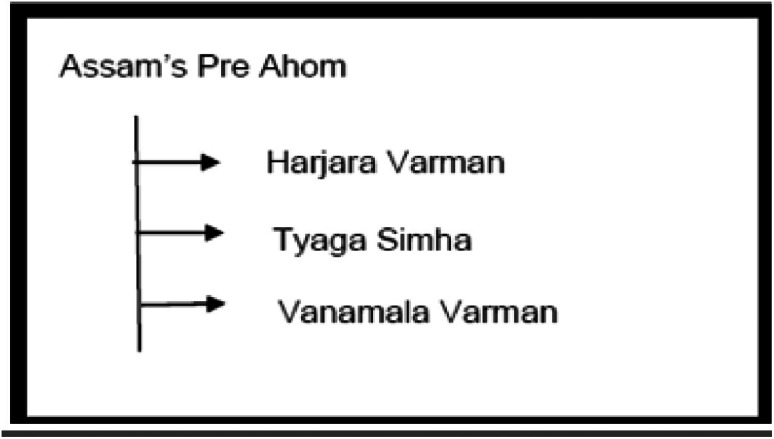
Structure of Assam’s Pre-Ahom type coin Dataset.

**Fig. 15:**
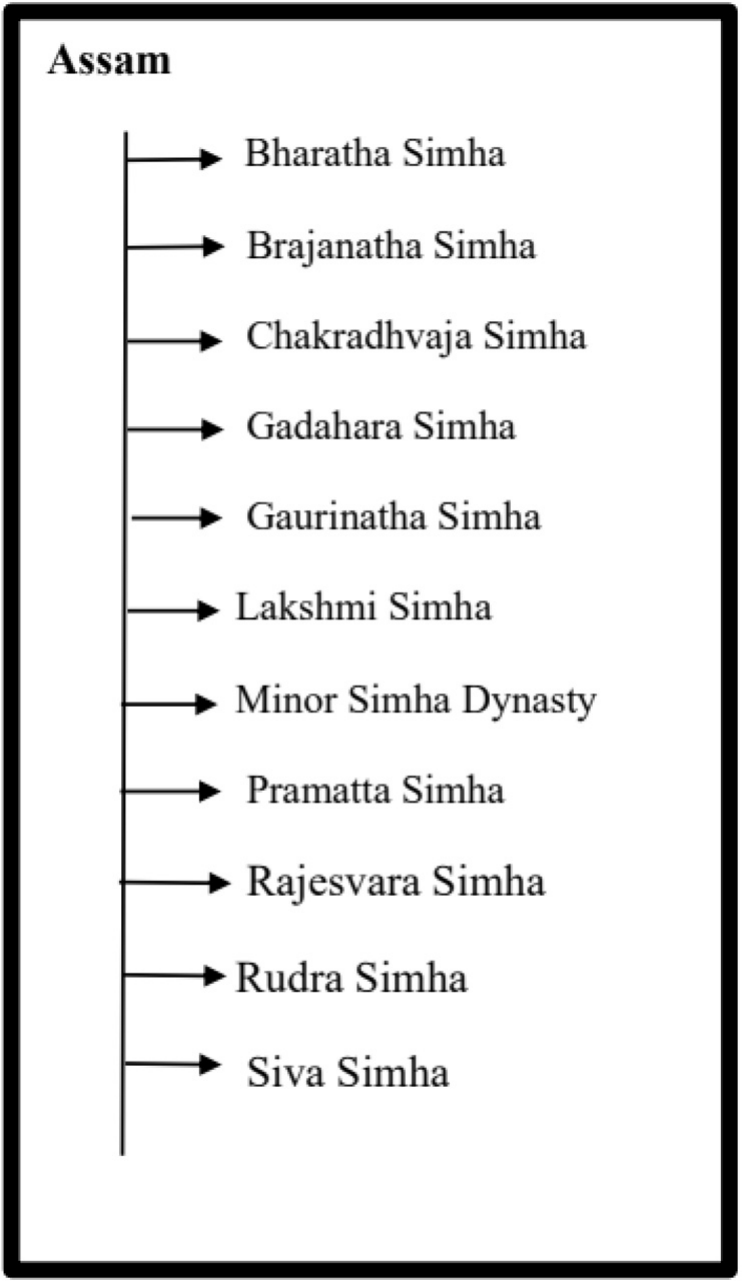
Structure of Assam type coin dataset.

**Fig. 16:**
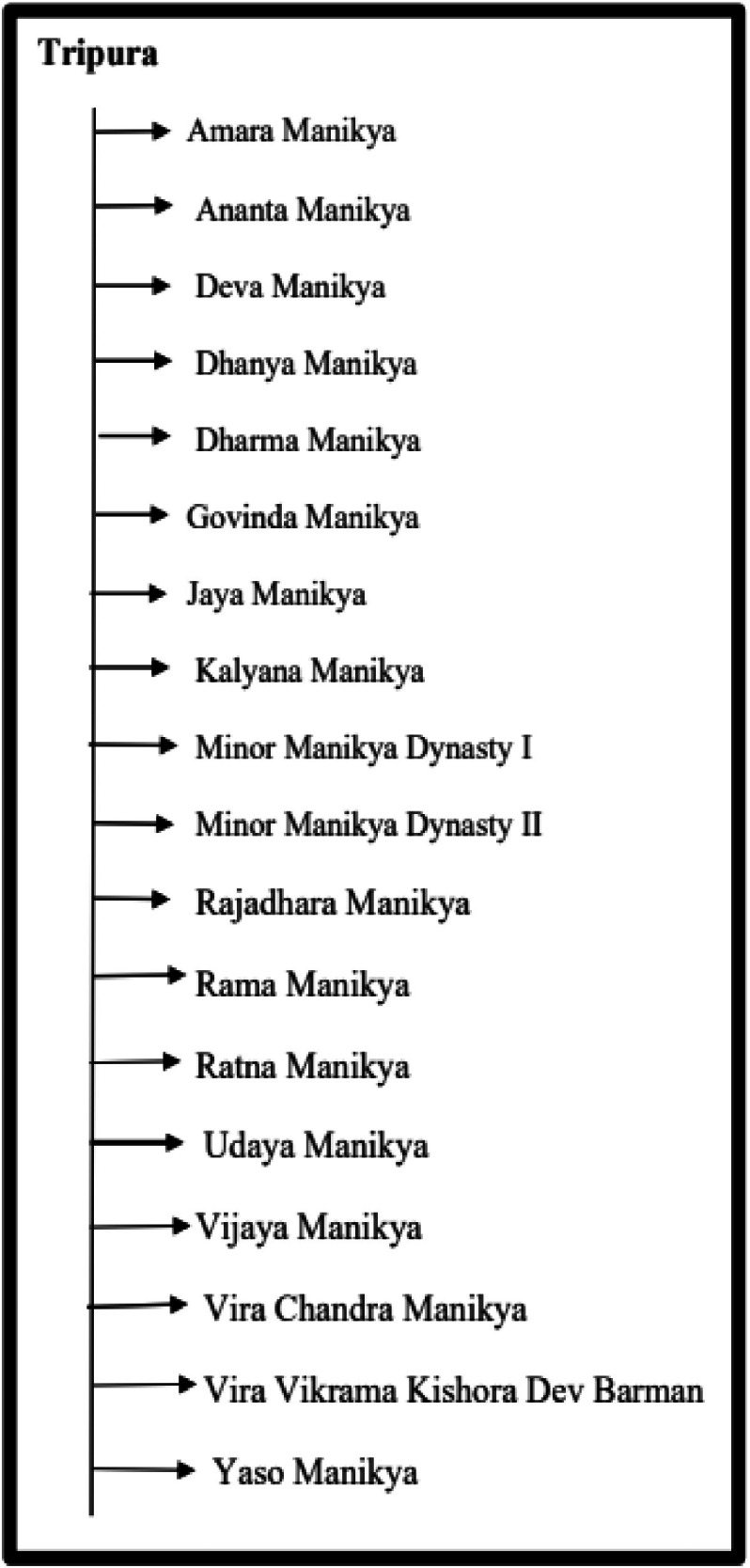
Structure of Tripura type coin dataset.

**Fig. 17:**
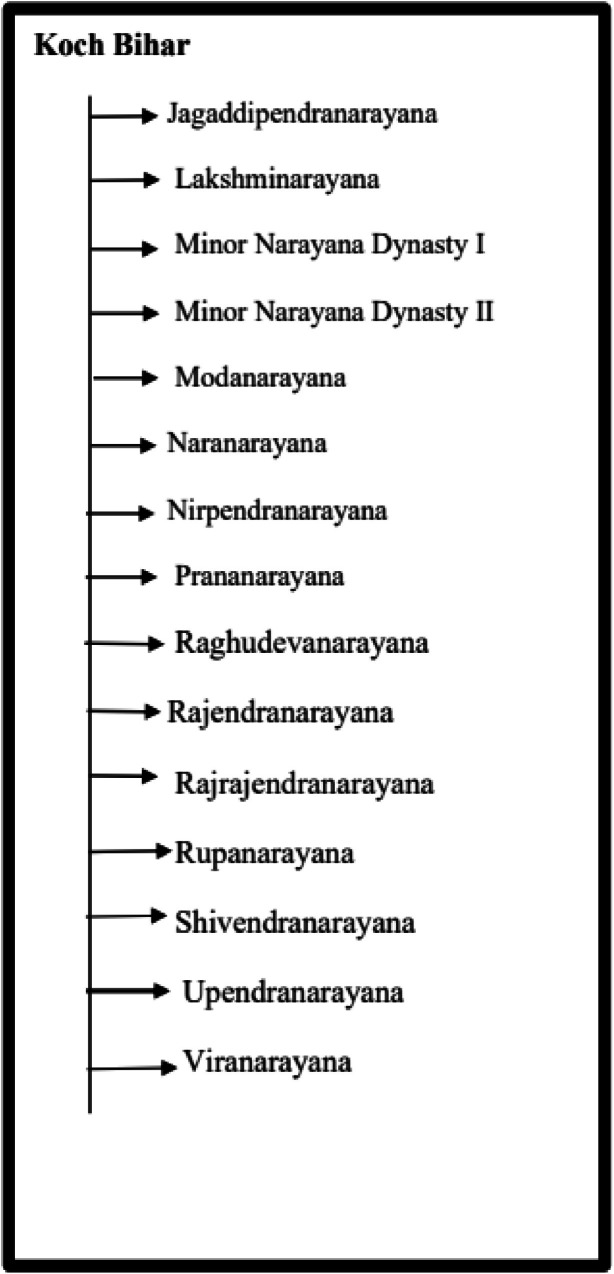
Structure of Koch Bihar type coin dataset.

**Fig. 18:**
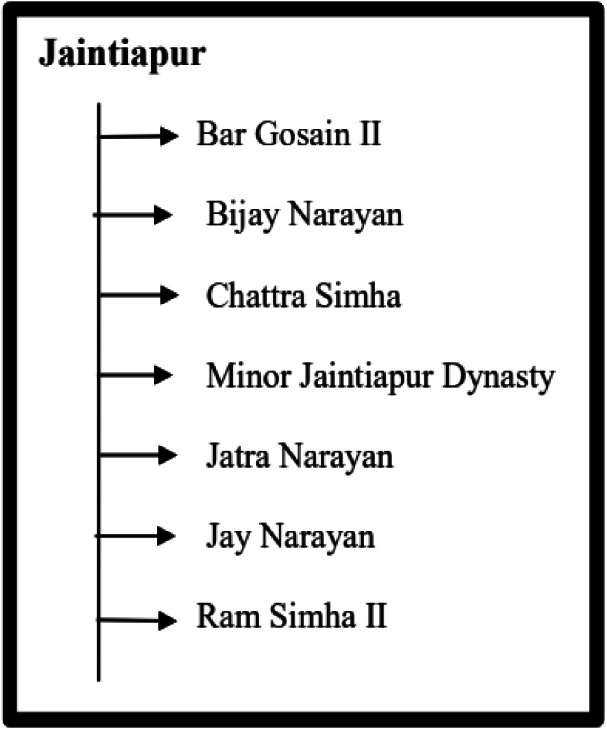
Structure of Jaintiapur type coin dataset.

The dataset is organized in a hierarchical structure, with folders and subfolders arranged according to region and historical classification. Data files are distributed across four main directories: Assam, Tripura, Koch Bihar, and Jaintiapur. Assam is divided into two distinct historical periods: the Pre-Ahom and Ahom periods. Fig. 13 provides an overview of the dataset, including a hierarchical organization of image folders, corresponding annotation files, along with the annotation format. Additionally, the class structure for each region, along with the corresponding dynasty and king names, is illustrated in Figs. 14–18.

Within each regional folder, images are organized by their specific dynasty or king. Additionally, there are some minor dynasty classes where minor kings’ images are located. Image filenames are assigned a unique serial number for each region, which directly corresponds to entries in the annotation files [16]. Additionally, the same hierarchical structure is maintained in the annotation files through the ‘Data Class’, ‘SL No.’, and ‘king’ columns, ensuring consistency between the folder organization and annotation labels. This structure enables straightforward mapping between image directories and their corresponding metadata for machine learning applications.

Annotation files are also distributed separately for each region and provided in both CSV and XLSX formats. Every single row in the annotation file contains information for a single coin image entry. The annotation file includes the following fields: serial number, acronym, source, reference, data class, king, year, denomination, weight, size, metal type, obverse motif, reverse motif, obverse inscription, reverse inscription, obverse circular inscription, and reverse circular inscription [16]. To ensure consistency and accuracy, all annotations were manually verified by numismatic experts and cross-checked against established numismatic references [1,[11], [12], [13], [14], [15]].

Fig. 14 illustrates the structure of Assam’s Pre-Ahom type coin dataset, which includes three kings. It provides a breakdown of how coin images are organized in groups according to the king.

Fig. 15, Fig. 16 illustrate the structures of the Assam and Tripura type coin datasets, both of which include multiple kings and show how the coin images are organized within each group. Moreover, for both of these regions, for Assam one and for Tripura two minor dynasty classes are included, where images from minor rulers are grouped into minor dynasty categories.

Fig. 17, Fig. 18 illustrate the structures of the Koch Bihar and Jaintiapur type coin datasets, showing how the coin images are organized for each group. Furthermore, Koch Bihar minor samples are divided into two minor classes based on their historical period. In contrast, Jaintiapur dataset contains one minor dynasty category for its regional coin images. The complete dataset is publicly available on the Mendeley Data repository (DOI: https://data.mendeley.com/datasets/275grzntd3/3) [16]. The repository contains all processed coin images in TIFF format as Data Files, along with corresponding annotation files in CSV and XLSX formats. The dataset is organized hierarchically by region and class, as described in the manuscript and illustrated in Fig. 13, Fig. 14, Fig. 15, Fig. 16, Fig. 17, Fig. 18. Users can directly download the dataset from the repository, extract the files, and use the annotation files to map images to their labels and metadata. To guide users regarding folder structure, file format, and usage, a detailed README.md file is also included in the repository. Additionally, copyright information from permission-granted auction houses is included as Supplementary Materials in the Mendeley data repository [16].

**Fig. 19:**
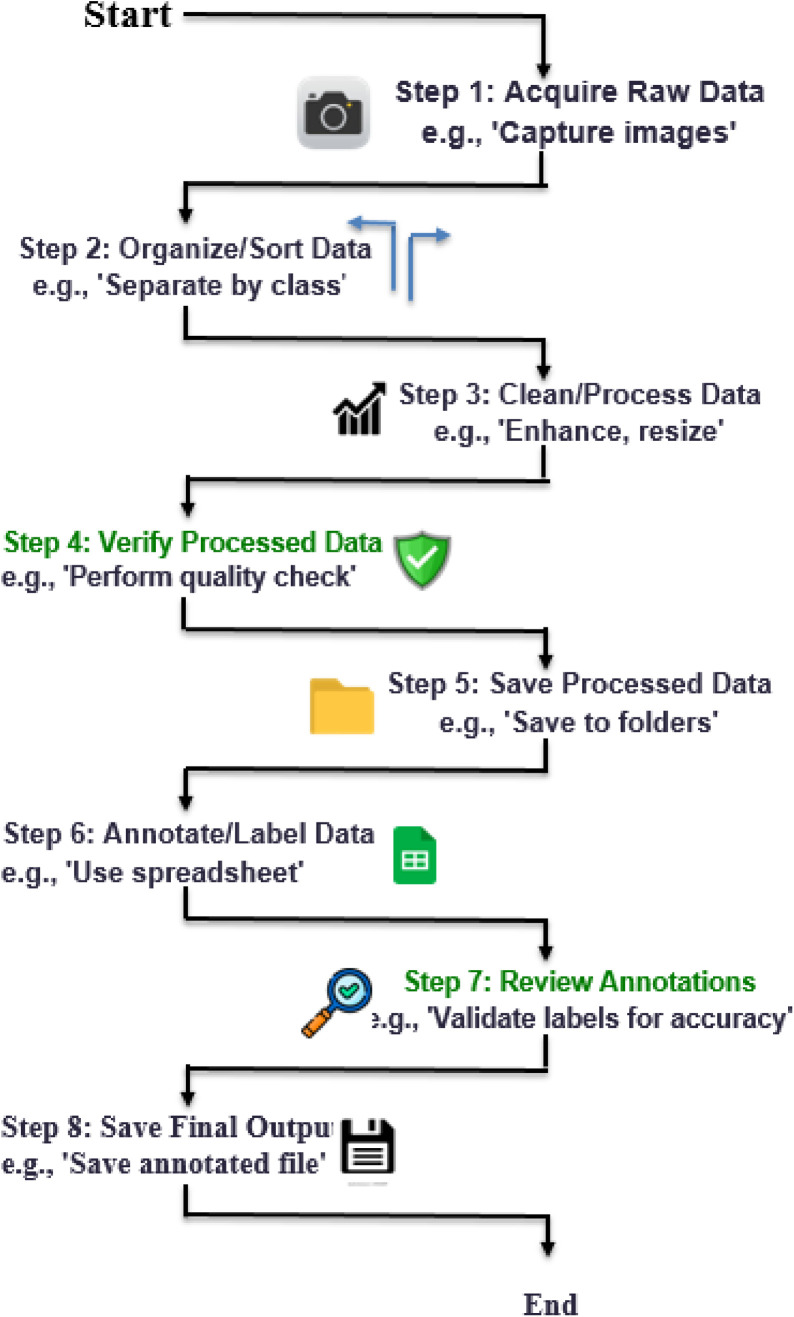
Workflow diagram of the complete dataset generation pipeline from image acquisition to public release.

Fig. 19 illustrates the complete workflow of the dataset generation pipeline, including acquisition, preprocessing, annotation, validation, and repository publication. Additionally, it provides a clear and reproducible pipeline for dataset construction.

Summary of Data Generation Pipeline

The complete data generation process followed a sequential and structured workflow:

1. Image Acquisition – Coin images were collected from private collections and five permission granted auction houses. For capturing private collection images, a standardized smartphone imaging setup was used.
2. Initial Screening – Images were carefully reviewed to exclude corrupted and unclear coin images that could not be confidently classified to specific region, dynasty, or class.
3. Class Organization – All the verified images were grouped according to their region and further organized by king-level classes, including some minor dynasty classes.
4. Image Pre-processing – Images underwent sequential preprocessing stages, where bilateral denoising, background normalization, resizing to 512×512 pixels using LANCZOS interpolation, and conversion to TIFF format were performed on all the organized images.
5. Manual Annotation – A structured manual annotation process was used to record metadata and create annotation files in CSV and XLSX formats based on established numismatic references.
6. Expert Validation – All class-assigning processes were verified using authoritative literature and validated by numismatic experts to ensure historical accuracy.
7. Repository Publication – Final processed images and annotation files in both formats, CSV and XLSX, were uploaded to the Mendeley Data repository for public access.

Supplementary materials include scripts for loading images and annotations, preprocessing, feature extraction, and dataset exploration, such as class distribution analysis and image consistency verification with annotations.

The photographic data were assigned suitable labels and annotations in accordance with the system of labelling and annotation framework illustrated in Fig. 19, where coins were categorized based on obverse motif design and coin weight. Some slight differences in these designs, called varieties, were also carefully considered during annotation. The obtained pictures from the internet and personal collections were categorized according to numismatic references [[11], [12], [13], [14], [15]] and confirmed by numismatic specialists. Labels were manually added through structured spreadsheet annotations based on established archaeological and numismatic sources. The dataset represents the historical context, cultural meaning, and visual information of each North-Eastern Indian coin [1]. The conservative nature of this approach ensures that the annotations are based on the historical and cultural aspects of the coins and offer a valid, genuine basis for classifying North-Eastern Indian coins. This improves the value of the dataset for machine learning applications, also maintaining consistency with established numismatic research standards.

## Limitations

The dataset is centered on the coins of North-Eastern India, given the difficulties posed by image scarcity. Since many historical coin types are extremely rare, the sample is not quite representative of the full gamut of numismatics of North-Eastern Indians. Cultural heritage incorporated into every coin of the North-Eastern Indian region makes the classification process more complex, as artistic differences and historical backgrounds are specific and, in some cases, impossible to describe exhaustively. The sample is limited to these coins and provides genuine, readily applicable data for analysis. It may perhaps not represent the complete range of North-Eastern Indian numismatics, as it may be limited to a small number of images. Although it is highly structured and professional, the dataset will be limited to this type of coin, and researchers must be careful when extrapolating the results to other coins.

## Ethics Statement

It should be emphasized that all of the pictures of the coin were either taken from five permission granted auction houses listed in the manuscript or from private collections. The usage of these coins is authorized by these auction houses. The procedure of data collection was conducted with a dedication to transparency and in correspondence with accepted ethical norms.

## CRediT author statement

**Ishtiak Al Mamoon:** Conceptualization, Data Validation, Investigation, Supervision, Funding acquisition, Writing –original draft; **Saddat Kabir:** Data Curation, Methodology, Software, Writing – original draft; **Ariful Islam:** Data curation, Methodology, Software, Visualization, Writing – original draft; **Golam Moula:** Data Curation, Methodology, Visualization; **Wafial Hasnat Laisa:** Data Curation, Methodology, Visualization; **Abu Sufian:** Data Curation, Methodology; **Md. Mahbub Hasan Akash:** Resources Software;**Sheekar Banerjee:** Writing-review & editing, Review; **Md. Samiul Alom:** Project administration; **Md. Abdul Awal:** Supervision, Review; **A.K.M. Muzahidul Islam:** Supervision, Review, Funding acquisition; **Utpal Kanti Das:** Supervision, Review.

## Data Availability

Mendeley DataNorth Eastern Indian Numismatics (Original data)

## Appendix Supplementary materials

Image, application 1
