# Supplementary material for: A novel dataset of North-Eastern Indian coins for machine learning-based classification
Source: Data Brief. 2026 May 1;66:112813. doi: 10.1016/j.dib.2026.112813 (PMC13195760; doi:10.1016/j.dib.2026.112813)
Supplement: Supplementary file 1 [file mmc1.zip › Supplymentary Materials/Devid Feldman Auction House.pdf]

Dr. Ishtiak Al Mamoon <ishtiak.cse@iubat.edu>

Looking for permission and obtaining a copy to publish coin images

Ishtiak Al Mamoon <ishtiak.cse@iubat.edu> Tue, Oct 21, 2025 at 5:13 PM
To: Info at David Feldman <info@davidfeldman.com>
Cc: Info Feldmanauctions <Info@feldmanauctions.com>

Dear Mr. Feldman,
I really appreciate your prompt answer and kind concern.
Best Regards,
Ishtiak Al Mamoon, Ph.D.

On 21 Oct, 2025, at 3:34 PM, Info at David Feldman <info@davidfeldman.com> wrote:

dear Sir,

Many thanks for your email.

This is perfectly fine.

Kind regards,

Joël Feldman
Managing Director | Directeur général
joel@davidfeldman.com
+41 (0) 22 727 07 77 | **+41 (0) 77 5218748**
Ch. du Pavillon 2, 1218 Le Grand-Saconnex, Switzerland
www.davidfeldman.com

IMPORTANT NOTICE: This e-mail message is intended to be received only by persons entitled to receive the confidential information it may contain. This e-mail message may contain information that is confidential and legally privileged. Please do not read, copy, forward, or store this message unless you are an intended recipient of it. If you have received this message in error, please forward it to the sender and delete it completely from your computer system.

From: Dr. Ishtiak Al Mamoon <ishtiak.cse@iubat.edu>
Sent: 21 October 2025 11:19 AM
To: Info Feldmanauctions <Info@feldmanauctions.com>
Subject: Looking for permission and obtaining a copy to publish coin images

I hope this message finds you well. I am writing this email to take permission for the use of images from your collections in our manuscripts on ancient Indian,medieval Bengal, Shasanka, North Eastern India and the samatata data set, which we are preparing to submit to the journal Data in Brief.

The journal's requirements for data accessibility and reusability necessitate that we obtain additional permissions. **Specifically, we need to ensure that the images can be freely reused by the readership of Data in Brief.**

We definitely cite your © David Feldman SA **auction house name and put copyright information on all images we collected from your auction house.** Could you kindly extend the permission to include the free reuse of these images by the journal's readership? This would involve allowing the images to be published under terms that

permit free access and reuse by others without any restrictions. We believe that the broader dissemination and potential reuse of these images will greatly benefit the academic community and further highlight the valuable collections held at your auction house. We would greatly appreciate your prompt attention to this matter and your assistance in helping us comply with the publication requirements.

I have purchase one jayanaga coin Lot: 1248 on silk road II Auction/

Thank you very much for considering our request. Please let me know if you need any additional information or if there are any issues we can help clarify.

Looking forward to your positive response.

Best Regards

Dr. Ishtiak Al Mamoon, SMIEEE
Associate Professor
Department of Computer Science and Engineering
College of Engineering and Technology (CEAT)
IUBAT—International University of Business Agriculture and Technology
4, Embankment Drive Road, (Off Dhaka-Ashulia Road)
Sector 10, Uttara Model Town, Dhaka-1230
H/P: +880-1713229860
