# Supplementary material for: A novel dataset of North-Eastern Indian coins for machine learning-based classification
Source: Data Brief. 2026 May 1;66:112813. doi: 10.1016/j.dib.2026.112813 (PMC13195760; doi:10.1016/j.dib.2026.112813)
Supplement: Supplementary file 1 [file mmc1.zip › Supplymentary Materials/Jain Auction House.pdf]

Dr. Ishtiak Al Mamoon <ishtiak.cse@iubat.edu>

Looking for permission and obtaining a copy to publish coin images for non commercial purposes

Sandip Jain <sjain2in@gmail.com> Tue, Jul 15, 2025 at 12:19 PM
To: Ishtiak Al Mamoon <ishtiak.cse@iubat.edu>

Your request is approved.

On Tue, 15 Jul 2025 at 11:31 AM, Ishtiak Al Mamoon <ishtiak.cse@iubat.edu> wrote:

Subject: Looking for permission and obtaining a copy to publish coin images for non commercial purposes

Dear Mr. Sandip Jain,
I hope this message finds you well. I am writing this email to take permission for the use of images from your collections in our manuscripts on mediaval Bengal, Shasanka, North Eastern India and the samatata data set, which we are preparing to submit to the journal Data in Brief.

The journal's requirements for data accessibility and reusability necessitate that we obtain additional permissions. **Specifically, we need to ensure that the images can be freely reused by the readership of Data in Brief.**

We definitely cite your ©Jain auction house name and put copyright information on all images we collected from your auction house. Could you kindly extend the permission to include the free reuse of these images by the journal's readership? This would involve allowing the images to be published under terms that permit free access and reuse by others without any restrictions. We believe that the broader dissemination and potential reuse of these images will greatly benefit the academic community and further highlight the valuable collections held at your auction house. We would greatly appreciate your prompt attention to this matter and your assistance in helping us comply with the publication requirements.

\Thank you very much Sandip da for considering our request. Please let me know if you need any additional information or if there are any issues we can help clarify.

Looking forward to your positive response.

Best Regards

Dr. Ishtiak Al Mamoon, SMIEEE
Associate Professor
Department of Computer Science and Engineering
College of Engineering and Technology (CEAT)
IUBAT—International University of Business Agriculture and Technology
4, Embankment Drive Road, (Off Dhaka-Ashulia Road)
Sector 10, Uttara Model Town, Dhaka-1230
H/P: +880-1713229860
