# Supplementary material for: A novel dataset of North-Eastern Indian coins for machine learning-based classification
Source: Data Brief. 2026 May 1;66:112813. doi: 10.1016/j.dib.2026.112813 (PMC13195760; doi:10.1016/j.dib.2026.112813)
Supplement: Supplementary file 1 [file mmc1.zip › Supplymentary Materials/Oswal auction House.pdf]

Gmail

Zakaria Shams Siam <zakarias@pu.edu.bd>

Re: Looking for permission and obtaining a copy to publish coin image —non commercial purposes

Dr. Ishtiak Al Mamoon <ishtiakm@pu.edu.bd> Wed, May 22, 2024 at 7:06 PM
To: girish veera <hgveera27@gmail.com>
Cc: Zakaria Siam <zakarias@pu.edu.bd>

Best Regards
Ishtiak Al Mamoon, Ph.D., SMIEEE
Associate Professor
ECE Department
Presidency University
Secretariat, ICBBDB 2021
TCP, MobiHealth 2021
H/p: +8801713229860
Email: i.a.mamoon@ieee.org
ishtiakm@pu.edu.bd

On 22 May, 2024, at 6:53 PM, girish veera <hgveera27@gmail.com> wrote:

Respected Dr. Ishtiak

Nice to know about your project.

We are granting permission to use our website images for your publication. As you mentioned please gives require credit "Oswal Auctions- Mumbai". Thanks.

Regards

Girish

Oswal Auctions Gala# 307, Narayan Udyog Bhavan,

Chiwda Galli, Lalbaug,

Mumbai- 400012

Mobile- 9324626548

On Tue, May 21, 2024 at 10:40 PM Dr. Ishtiak Al Mamoon <ishtiakm@pu.edu.bd> wrote:

Dear Mr. Girish Veera
I hope this message finds you well. I am writing this email to take permissions granted for the use of images from your collections in our manuscripts on North Eastern India, Shasanka and samatata data set, which we are preparing to submit to the journal Data in Brief.

The journal's requirements for data accessibility and reusability necessitate that we obtain additional permissions. **Specifically, we need to ensure that the images can be freely reused by the readership of Data in Brief.**

We definitely site your auction house name and put copyright information all image we collected from your auction house.

Could you kindly extend the permission to include the free reuse of these images by the journal's readership? This would involve allowing the images to be published under terms that permit free

access and reuse by others without any restrictions.

We believe that the broader dissemination and potential reuse of these images will greatly benefit the academic community and further highlight the valuable collections held at your auction house.

We would greatly appreciate your prompt attention to this matter and your assistance in helping us comply with the publication requirements.

Thank you very much for considering our request. Please let me know if you need any additional information or if there are any issues we can help clarify.

Looking forward to your positive response.

Best Regards

Ishtiak Al Mamoon, Ph.D., SMIEEE
Associate Professor
ECE Department
Presidency University
Secretariat, ICBBDB 2021
TCP, MobiHealth 2021
H/p: +8801713229860
Email: i.a.mamoon@ieee.org
ishtiakm@pu.edu.bd
