# Supplementary material for: A novel dataset of North-Eastern Indian coins for machine learning-based classification
Source: Data Brief. 2026 May 1;66:112813. doi: 10.1016/j.dib.2026.112813 (PMC13195760; doi:10.1016/j.dib.2026.112813)
Supplement: Supplementary file 1 [file Classical Numesmatic Gallery.pdf › Supplymentary Materials/Classical Numesmatic Gallery.pdf]

Gmail Zakaria Shams Siam <zakarias@pu.edu.bd>

Fwd: Looking for permission and obtaining a copy to publish coin image —non commercial purposes

Dr. Ishtiak Al Mamoon <ishtiakm@pu.edu.bd> Tue, May 7, 2024 at 11:07 PM
To: Zakaria Siam <zakarias@pu.edu.bd>

Best Regards
Ishtiak Al Mamoon, Ph.D., SMIEEE
Associate Professor
ECE Department
Presidency University
Secretariat, ICBBDB 2021
TCP, MobiHealth 2021
H/p: +8801713229860
Email: i.a.mamoon@ieee.org
ishtiakm@pu.edu.bd

Begin forwarded message:

From: CNG CNG <cng@cngcoins.com>
Date: 18 April, 2024 8:22:38 PM GMT+6
To: "Dr. Ishtiak Al Mamoon" <ishtiakm@pu.edu.bd>
Subject: Re: Looking for permission and obtaining a copy to publish coin image —non commercial purposes

Dear Dr. Al Mamoon,

Thank you for your e-mail.
Yes, you have our permission to use our photos as long as you cite Classical Numismatic Group, LLC, along with our website, as the source.

Best Regards,

Kate Rill
Classical Numismatic Group, LLC
PO Box 479
Lancaster PA 17608-0479 USA
Phone: 717-390-9194 Fax: 717-390-9978
Email: cng@cngcoins.com Website: http://www.cngcoins.com

On Apr 18, 2024, at 9:33 AM, Dr. Ishtiak Al Mamoon <ishtiakm@pu.edu.bd> wrote:

Dear concerned person,
I am an artificial intelligence researcher, very recently we are working on North Eastern India and Bengal king Sashanka's classification and data set development. Furthermore, we are planning to submit manuscripts in The Elsevier journal Data in Brief. In these manuscripts, we are using a good number of coin images from your archive . And I can assure you that no alternative was found during acquisition . The title of manuscripts as follows :

A Novel Dataset of North Eastern Indian Coins for Machine Learning-Based Classification.

Numismatic Dataset of First Independent Bengal King Sashanka

The journal authorities are asking for copyright permission from your auction house. Please consider this is not **commercial activity and only limited to research purpose**s.

I will be very much obliged if you kindly give us permission to use your coin images by issuing a copyright certificate.
Thanks in advance

Best Regards,

Ishtiak Al Mamoon, Ph.D., SMIEEE
Associate Professor
Department of Computer Engineering
Presidency University, Baridhara Campus Email: ishtiakm@pu.edu.bd
H/P: +880-171-3229860
