# Supplementary material for: A novel dataset of North-Eastern Indian coins for machine learning-based classification
Source: Data Brief. 2026 May 1;66:112813. doi: 10.1016/j.dib.2026.112813 (PMC13195760; doi:10.1016/j.dib.2026.112813)
Supplement: Supplementary file 1 [file Marudhar Arts.pdf › Supplymentary Materials/Marudhar Arts.pdf]

Gmail

Zakaria Shams Siam <zakarias@pu.edu.bd>

Looking for permission and obtaining a copy to publish coin image —non commercial purposes

Dr. Ishtiak Al Mamoon <ishtiakm@pu.edu.bd> Mon, May 13, 2024 at 4:43 PM
To: Marudhar Arts <info@marudhararts.com>
Cc: Zakaria Siam <zakarias@pu.edu.bd>

Attn to :

Maru Rajender
Auctioneer | Editor | Organizer | Director
www.marudhararts.com
Marudhar Arts
85 M. G. Road,
Next to Barton Center,
Bangalore 560 001 (INDIA)

Dear Mr Maru Rajendar,

I hope this message finds you well. I am writing to follow up on the previous permissions granted for the use of images from your collections in our manuscripts on North Eastern Indian, Ancient Bengal, Shasanka and samatata data set, which we are preparing to submit to the journal Data in Brief.

We are grateful for your support and the permission you have already granted for the use of these images in our research. However, the journal's requirements for data accessibility and reusability necessitate that we obtain additional permissions. **Specifically, we need to ensure that the images can be freely reused by the readership of Data in Brief.**

Could you kindly extend the permission to include the free reuse of these images by the journal's readership? This would involve allowing the images to be published under terms that permit free access and reuse by others without any restrictions.

We believe that the broader dissemination and potential reuse of these images will greatly benefit the academic community and further highlight the valuable collections held at your institution.

We would greatly appreciate your prompt attention to this matter and your assistance in helping us comply with the publication requirements.

Thank you very much for considering our request. Please let me know if you need any additional information or if there are any issues we can help clarify.

Looking forward to your positive response.
Best Regards,

Ishtiak Al Mamoon, Ph.D., SMIEEE
Associate Professor
Department of Electrical and Computer Engineering
Presidency University, Baridhara Campus
Email: ishtiakm@pu.edu.bd
H/P: +880-171-3229860

[Quoted text hidden]

Gmail

Zakaria Shams Siam <zakarias@pu.edu.bd>

Looking for permission and obtaining a copy to publish coin image —non commercial purposes

Marudhar Arts <info@marudhararts.com> Mon, Apr 15, 2024 at 3:39 PM
To: "Dr. Ishtiak Al Mamoon" <ishtiakm@pu.edu.bd>
Cc: Zakaria Siam <zakarias@pu.edu.bd>

Subject: Proper Usage of Images from Marudhar Arts® Auctions in Academic Work

Dear Professor,

We are pleased to grant you and your students permission to utilize images from Marudhar Arts® Auctions for academic purposes. We value the contribution of academic research and are glad to support your endeavors.

However, we kindly request that you and your students adhere to the following guidelines to ensure proper usage and crediting of our images:

1. Please use the correct name of our firm, Marudhar Arts® Auctions, along with the copyright logo and city, as mentioned in our previous correspondence. It is crucial to avoid misspelling our name. It has to be strictly used like this - **© Marudhar Arts, India**

2. While we encourage the use of the data we provide, we expect proper credits to be given for our hard work in every research paper where our images are utilized.

By following these guidelines, you contribute to maintaining the integrity of our brand and acknowledge the effort we put into curating and providing valuable data.

Thank you for your understanding and cooperation.

Best Regards
Maru Rajender
Auctioneer | Editor | Organizer | Director

www.marudhararts.com

https://www.facebook.com/MarudharArts/

Marudhar Arts
85 M. G. Road,
Next to Barton Center,
Bangalore 560 001 (INDIA)

+91 - 88670-29800 **(10AM to 6PM IST)**

[Quoted text hidden]

Gmail

Zakaria Shams Siam <zakarias@pu.edu.bd>

Looking for permission and obtaining a copy to publish coin image —non commercial purposes

Marudhar Arts <info@marudhararts.com>
To: "Dr. Ishtiak Al Mamoon" <ishtiakm@pu.edu.bd>
Cc: Zakaria Siam <zakarias@pu.edu.bd>

Wed, May 15, 2024 at 11:23 AM

Dear Sir,

Good morning.

As discussed last time, as long as every image is duly acknowledged whenever it is reused or republished for any purpose with due credits given to each image as **- © Marudhar Arts, India,** you are allowed to use it.

Requesting you to share the Journal which you have mentioned last time.

Thank you

Best Regards
Maru Rajender
Auctioneer | Editor | Organizer | Director

www.marudhararts.com

https://www.facebook.com/MarudharArts/

Marudhar Arts
85 M. G. Road,
Next to Barton Center,
Bangalore 560 001 (INDIA)

+91 - 88670-29800 **(10AM to 6PM IST)**

[Quoted text hidden]
